# T Cell Inactivation by Poxviral B22 Family Proteins Increases Viral Virulence

**DOI:** 10.1371/journal.ppat.1004123

**Published:** 2014-05-15

**Authors:** Dina Alzhanova, Erika Hammarlund, Jason Reed, Erin Meermeier, Stephanie Rawlings, Caroline A. Ray, David M. Edwards, Ben Bimber, Alfred Legasse, Shannon Planer, Jerald Sprague, Michael K. Axthelm, David J. Pickup, David M. Lewinsohn, Marielle C. Gold, Scott W. Wong, Jonah B. Sacha, Mark K. Slifka, Klaus Früh

**Affiliations:** 1 Vaccine and Gene Therapy Institute, Oregon National Primate Research Center, Portland, Oregon, United States of America; 2 Division of Neuroscience, Oregon National Primate Research Center, Portland, Oregon, United States of America; 3 Department of Pulmonary and Critical Care Medicine, Oregon Health & Science University, Portland, Oregon, United States of America; 4 Portland Veterans Administration Medical Center, Portland, Oregon, United States of America; 5 Department of Molecular Genetics and Microbiology, Duke University Medical Center, Durham, North Carolina, United States of America; 6 Division of Pathobiology and Immunology, Oregon National Primate Research Center, Portland, Oregon, United States of America; Fox Chase Cancer Center, United States of America

## Abstract

Infections with monkeypox, cowpox and weaponized variola virus remain a threat to the increasingly unvaccinated human population, but little is known about their mechanisms of virulence and immune evasion. We now demonstrate that B22 proteins, encoded by the largest genes of these viruses, render human T cells unresponsive to stimulation of the T cell receptor by MHC-dependent antigen presentation or by MHC-independent stimulation. In contrast, stimuli that bypass TCR-signaling are not inhibited. In a non-human primate model of monkeypox, virus lacking the B22R homologue (MPXVΔ197) caused only mild disease with lower viremia and cutaneous pox lesions compared to wild type MPXV which caused high viremia, morbidity and mortality. Since MPXVΔ197-infected animals displayed accelerated T cell responses and less T cell dysregulation than MPXV US2003, we conclude that B22 family proteins cause viral virulence by suppressing T cell control of viral dissemination.

## Author Summary

We discovered that the largest gene in the genome of monkeypox viruses and several related viruses, including the virus causing smallpox, but not vaccine strains, encode a protein (B22) that renders the cellular immune system non-responsive. A particularly novel aspect of this work is that B22 proteins directly disable cells of the immune system as compared to previously known molecular strategies that help viruses to hide from the immune system. We further show that monkeypox viruses containing this protein are much more virulent in non-human primates than viruses that lack B22. Our observations suggest that B22 proteins contribute to monkeypox virulence and might have contributed to the severe disease manifestations of variola major virus. However, these data also suggest that B22 proteins could potentially be used to curb undesired immune responses such as autoimmunity or graft versus host disease.

## Introduction

Smallpox was among the deadliest infectious diseases in history and its eradication is a landmark in medicine. However, loss of orthopoxvirus (OPXV)-specific immunity facilitates the accidental introduction of zoonotic OPXV such as monkeypox virus (MPXV) and cowpox virus (CPXV) which cannot be eradicated due to animal reservoirs. This risk became evident during the first MPXV outbreak outside Africa, which occurred in the US in 2003 [1]. Although MPXV does not spread efficiently by human-to-human contact it shares several key features of pathogenesis with variola virus (VARV) the causative agent of smallpox. MPXV is endemic in African rain forests with strains circulating in Central versus West Africa falling into two genetically distinct clades [2]. The West African clade, including US2003 strains, is considered less virulent based on *in vivo* studies conducted in cynomolgus monkeys, prairie dogs, and ground squirrels [3], [4], [5], [6]. Nevertheless, life-threatening disease was identified during the U.S. outbreak [1], [7].

The DNA genomes of OPXV encode approximately 200 open reading frames (ORFs) with around 90 highly conserved genes encoded in the central regions of the genome whereas the terminally coded genes vary among different OPXV and are responsible for differences in host range, virulence, and immune evasion [8]. Conserved genes among OPXV are highly related to each other resulting in cross-protection, i.e. prior infection with any one of the OPXV generally protects against serious disease by other OPX, so that vaccinia virus (VACV) is broadly protective against all OPXV. Protection against OPXV is remarkably long lived. During the 2003 MPXV outbreak, the number of lesions in previously vaccinated individuals was significantly lower with some individuals being completely protected from MPXV-associated disease [9]. Antibody (Ab) titers to the vaccine remain remarkably stable over the life of vaccinated individuals [10] and vaccine-mediated protection of non-human primates (NHP) against lethal MPXV challenge is Ab-mediated [11]. Similarly, vaccinated mice succumb to lethal challenge with mousepox ectromelia virus (ECTV) in the absence of Ab, despite the presence of poxvirus-specific T cells [12]. In contrast, T cells promote survival of vaccinated mice challenged with lethal doses of VACV [13], [14].

The limited role of T cells in protecting against virulent OPXV is surprising given that OPXV induce a strong T cell response recognizing multiple conserved epitopes [15]. Moreover, VACV is widely used as T cell-inducing vaccine vector [16], [17]. The reduced ability of T cells to control OPXV might, in fact, be directly related to virulence since T cells do limit virulence of CPXV provided that two gene products interfering with MHC-I antigen presentation were deleted [18]. Thus, the inability of T cells in protecting against virulent OPXV might be due to T cell evasion mechanisms.

In the case of CPXV, T cell evasion is mediated by two gene products that each interferes with different steps of the MHC-I antigen presentation pathway. CPXV203 binds to and retains MHC-I in the endoplasmic reticulum (ER) [19]. CPXV12 inhibits TAP-dependent peptide translocation across the ER membrane [18], [20]. MPXV contains a CPXV203 orthologue, but does not seem to retain MHC-I [21]. Instead, MPXV inhibits T cell activation by either MHC-dependent or by MHC-independent stimulation [21]. Thus, MPXV encodes one or more gene products that render T cells non-responsive.

Here, we identify the gene product responsible for this T cell inactivation as MPXV197, the largest gene in the MPXV genome. This predicted transmembrane protein belongs to the B22 family of proteins found in several OPXV including CPXV, ECTV and VARV, but not in VACV. We demonstrate that MPXV197 and related proteins of CPXV and VARV inactivate T cells by a novel mechanism. We further show that deletion of MPXV197 severely attenuates MPXV and prevents lethal disease in rhesus macaques (RM). Despite a substantial reduction of viral titers, RM infected with MPXV197-deleted virus had stronger and more rapid T cell responses consistent with B22 proteins contributing to OPXV virulence by suppressing T cell responses.

## Results

### MPXV197 is essential for T-cell inactivation by MPXV

**Figure 1:**
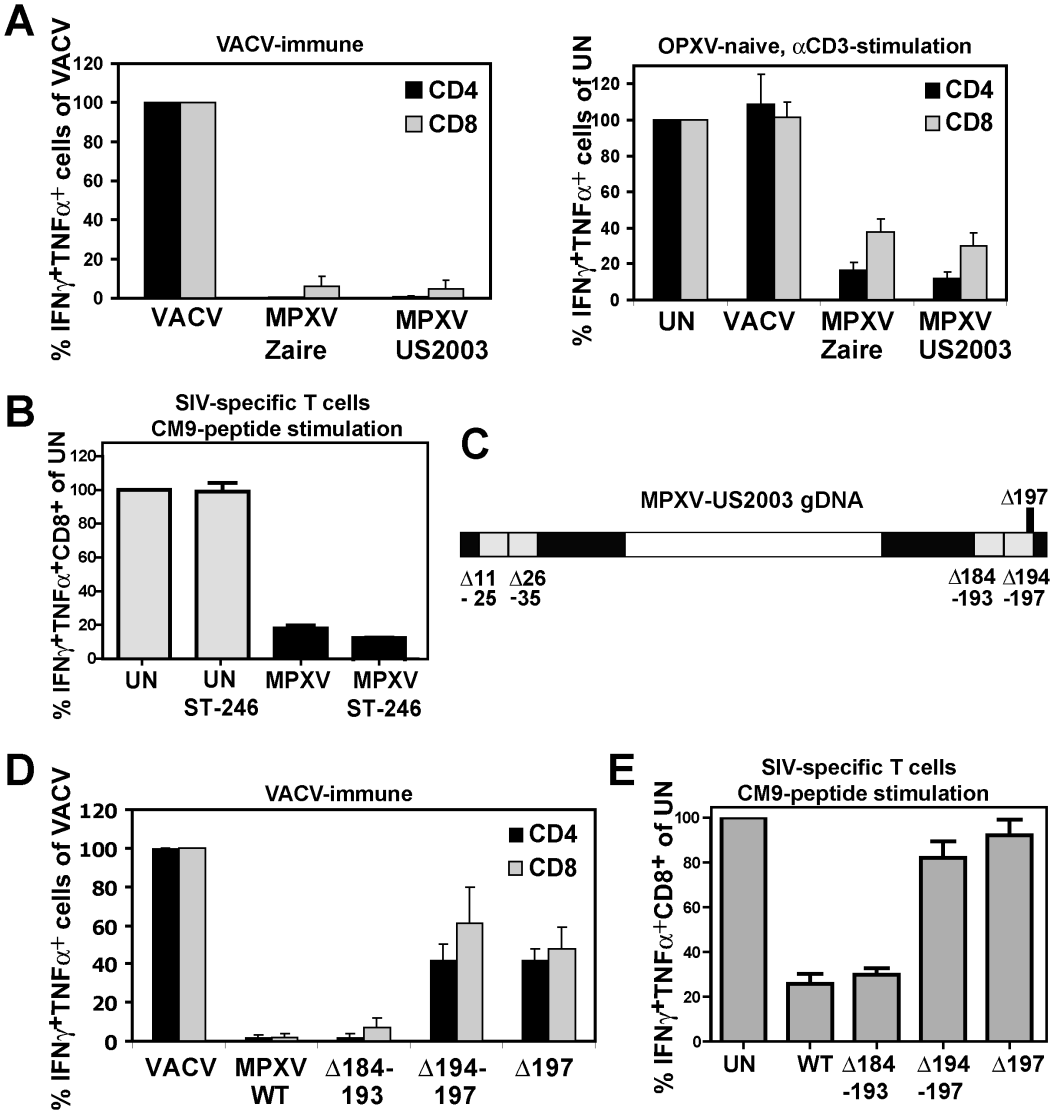
MPXV197 is required for T cell inhibition by MPXV. **A)** Left Panel: PBMC from VACV-immune subjects (n = 4) were infected with VACV, MPXV Zaire or MPXV US2003 (MOI of 0.5) for 18 h. Poxvirus-specific CD4+ and CD8+ T cell responses were measured by ICCS. Results are normalized to % of VACV-specific response. Right Panel: PBMC from VACV-naïve subjects (n = 3) were infected with indicated viruses or uninfected (UN) and T cells were stimulated with plate-bound αCD3 Ab for 6 h. **B)** CM9-specific RM CD8+ T-cells were incubated with HFF infected with MPXV US2003 (MOI of 2) in the presence or absence of 10 µM ST246 for 18 h prior to stimulation with CM9-peptide pulsed BLCL cells for 6.5 h. The percentage of IFNγ+TNFα+ cells as measured by ICCS is shown. **C)** Map of 10 Kb deletions (light grey) or a single ORF 197 deletion (black) in the terminal regions of the MPXV US2003 genome (black). **D)** Human CD4+ and CD8+ T cell responses to MPXV deletion mutants were determined by ICCS as in A). Infection rates of CD14+ monocytes in PBMC for MPXV US2003, MPXVΔ184-193, MPXVΔ194-197, and MPXVΔ197 were 73%, 81%, 65%, and 70%, respectively. **E)** Inhibition of CM9-specific CD8+ T-cell stimulation by MPXV US2003 or deletion mutants was measured by ICCS as in B). T cells were co-incubated with HFF cells infected with indicated viruses (MOI of 2, 10 µM ST246) for 18 h and then stimulated with CM9-peptide pulsed BLCLs for 6.5 h.

We previously demonstrated that MPXV (Zaire strain) inhibits CD4+ and CD8+ T-cell activation by both MHC-dependent and MHC-independent stimuli [21]. In contrast, T cell evasion by CPXV seemed to rely predominantly on inhibition of MHC-I-dependent antigen presentation by CPXV12 and CPXV203 [18], [19], [20], [22]. Since MPXV Zaire encodes an orthologue of CPXV203 we wanted to determine whether it is required for T cell evasion. The West-African strain MPXV US2003 lacks most of the CPXV203 orthologue [2]. We compared poxvirus-specific T cell responses elicited by MPXV Zaire and US2003 by infecting human PBMC from recently VACV-vaccinated donors with MPXV at an MOI of 0.3 and analyzed T cell responses by intracellular cytokine staining (ICCS) for TNFα+ IFNγ+ cells. VACV was used as control since it does not inhibit T cell stimulation [21]. VACV vigorously stimulated virus-specific IFNγ^+^TNFα^+^ CD4+ and CD8+ T cells whereas ≤6% of either response occurred with MPXV Zaire or MPXV US2003 (Fig. 1A). Total IFNγ^+^ T cell responses or total TNFα^+^ T cell responses were reduced to the same level as that observed with IFNγ^+^TNFα^+^ T cells (data not shown). This lack of T cell stimulation was not due to lack of cross-reactivity or reduced rates of infection since VACV-specific T-cells recognize cells infected with UV-inactivated MPXV[21] and MPXV and VACV infected comparable numbers of cells in PBMC (data not shown). Thus, the MPXV homologue of CPXV203 is not required for inhibition of poxvirus-specific T cells. To further examine whether the previously reported MHC-independent activation of T cells was also inhibited in the absence the CPXV203 orthologue we compared T cell activation by plate-bound αCD3 Ab in the presence of MPXV Zaire and US2003. As shown in Fig. 1A (right panel), MPXV US2003 retained the capability to inhibit MHC- independent T cell stimulation. T cell inhibitory genes are thus conserved in both clades of MPXV but absent or non-functional in VACV.

Since in PBMC, OPXV infect CD14+ monocytes but rarely T cells we concluded that T cell interference by MPXV is due to a *trans*-inhibition [21]. However, to rule out with certainty that MPXV inhibits T cells *in cis* we separated MPXV-infection from antigen presentation by infecting human foreskin fibroblasts (HFF) with MPXV and co-incubating these cells with rhesus macaque (RM)-derived T cell lines specific for the MaMu-A*01-restricted SIV GAG_181-189_ epitope CM9 [23]. As APC we used autologous B cells immortalized by simian lymphocryptovirus (BLCLs) [23]. Thus, in this assay the infected cells (HFF) do not contribute to T cell stimulation which is provided by peptide-pulsed BLCLs. To prevent dissemination of the virus from HFF to T cells we took advantage of the fact that compound ST-246 inhibits egress of viral particles from infected cells [24]. Control experiments demonstrated that ST246 efficiently (∼90%) prevented spread of VACV, CPXV, and MPXV to Jurkat T cells (Fig. S1). When ST-246-pretreated HFF were infected with MPXV, T cell stimulation by CM9 peptide-pulsed BLCLs was still inhibited to <10% of the uninfected cell control (Fig. 1B) confirming that MPXV inhibits T cell activation *in trans*. Since the T cell inhibitory factor is not secreted [21] this process most likely involves cell to cell contact.

To identify the T cell evasion gene(s) we generated four deletion mutants each lacking about 10 kb in the termini of the MPXV US2003 genome (Fig. 1C; Fig. S2A). Each of the mutants was examined for its ability to inhibit stimulation of T cells in PBMC from VACV-immune subjects (Fig. 1D) or peptide-stimulation of CM9-specific T cells from RM (Fig.1E). Mutants lacking ORFs 11–25, 26–35 (data not shown), or 184–193 did not activate poxvirus-specific T cells (Fig. 1D) and still inhibited peptide-stimulation of CM9-specific T cells (Fig. 1E). In contrast, MPXV lacking ORFs 194–197 activated both CD4+ and CD8+ T cells in VACV-immune PBMC (Fig. 1D) and no longer inhibited peptide stimulation of CM9-specific T cells (Fig. 1E). These data suggested that the MPXV194–197 genomic region encodes the T cell inhibitor. Since it seemed likely that T cell evasion would be mediated by a membrane bound extracellular protein, we deleted MPXV197 which is predicted to encode a large TM protein. As shown in Fig. 1D and E, MPXVΔ197 stimulated poxvirus-specific CD4+ and CD8+ T cells similar to VACV and peptide stimulation of CM9-specific T cells was no longer inhibited. Therefore, we conclude that MPXV197 is essential for T cell inhibition by MPXV.

### MPXV197 is sufficient to interfere with T-cell stimulation by multiple stimuli

**Figure 2:**
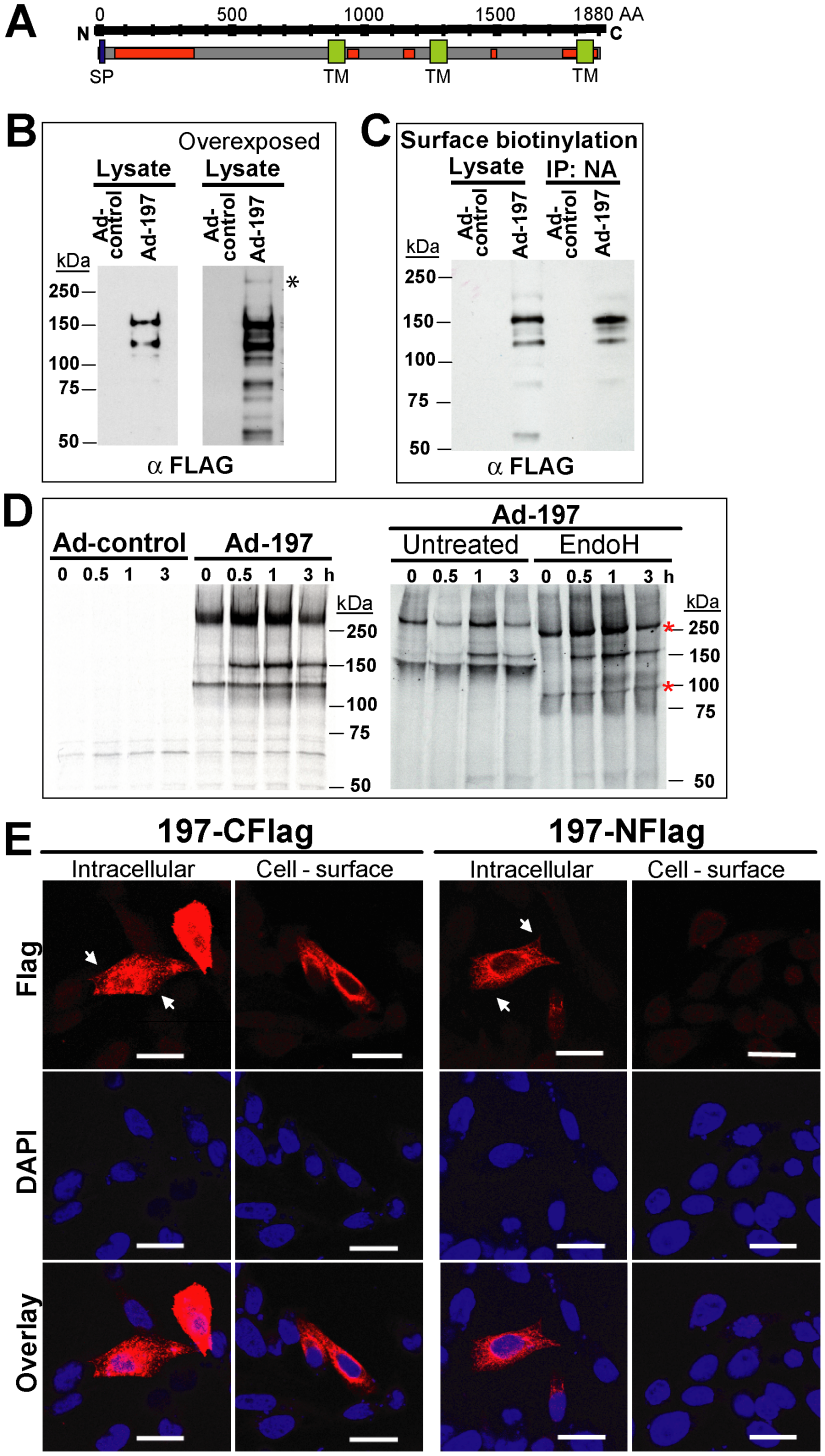
Molecular characterization and sub-cellular localization of MPXV197. **A)** Schematic representation of MPXV 197 with predicted signal peptide (SP, blue, SignalP), transmembrane domains (TM, green, TMPred), and N-linked glycosylation sites (red, NetNGlyc 1.0). **B)** CHO cells were transduced with Ad-197/Ad-tTA (‘Ad-197’) or Ad-tTA only (‘Ad-control’) for 24 hours and lysed in sample buffer prior to electrophoretic separation and immunoblotting with αFLAG. Right panel: Overexposure reveals a >250 kDa band (asterisk). **C)** CHO cells were transduced as in B). After 24 h, cell surface proteins were biotinylated followed by immunoprecipitation with NeutrAvidin, electrophoretic separation and immunoblotting with αFLAG. **D)** 24 h after transduction with the indicated expression vectors, CHO cells were metabolically labeled for 45 min followed by chase for 0.5, 1, and 3 h. Cell lysates were immunoprecipitated with αFLAG. In the right panel, samples were treated with EndoH or left untreated prior to electrophoretic separation. EndoH sensitive proteins are indicated by asterisks. **E)** Sub-cellular localization of C- and N-terminal FLAG fusions of MPXV197 was determined by IFA using αFLAG. CHO cells were either permeabilized (‘Intracellular’) or non-permeabilized (‘Cell-surface’) prior to IFA. Scale bar is 20 µm. Arrows indicate the plasma membrane.

To determine whether ectopically expressed MPXV197 inhibits T cell stimulation, we inserted a codon-optimized version into plasmid and adenovirus expression vectors. MPXV197 is the largest ORF in the genome of MPXV encoding for 1880 amino-acids with a predicted molecular mass of 212 kDa, a predicted cleavable N-terminal signal peptide (SP), multiple N-glycosylation sites, a C-terminal transmembrane (TM) domain, and potentially one or more internal TM domains (Fig. 2A) [2]. Transient expression of MPXV197 in CHO cells and immunoblotting with αFLAG-Ab revealed two predominant bands with apparent molecular mass of ∼150 kDa, and ∼140 kDa and several minor, smaller bands as well as a large protein >250 kDa (Fig. 2B). To determine which of these proteins were located at the cell surface we performed surface biotinylation followed by streptavidin-precipitation and immunoblot with αFLAG antibody. The ∼150 kDa species was the predominant species in this assay (Fig. 2C). Pulse-chase labeling revealed that the ∼150 kDa protein was a processing product derived from the large >250 kDa precursor protein. A minor ∼140 kDa fragment carrying the C-terminal Flag-tag was synthesized simultaneously with the large precursor protein suggesting that this fragment is derived from an internal start site (Fig. 2D). Interestingly, Endoglycosidase H (EndoH) –treatment reduced the apparent molecular mass of the largest and the smaller fragment whereas the ∼150 kDa processing product was EndoH-resistant. This result suggests that the full-length protein is processed into a ∼150 kDa fragment that is transported beyond the ER to the cell surface consistent with the surface biotinylation result. The C-terminal location of the FLAG-tag identifies the ∼150 kDa fragment as a C-terminal fragment. We further determined the sub-cellular localization of MPXV197 by immunofluorescence analysis (IFA) using confocal laser scanning microscopy (CLSM). Staining with αFLAG Ab of permeabilized or non-permeabilized CHO cells revealed that the C-terminus of MPXV197 locates to the extracellular face of the plasma membrane (Fig. 2E). In contrast, N-terminally Flag-tagged MPXV reacted with αFLAG Ab only when cells were permeabilized (Fig. 2E) consistent with the full-length protein and potential N-terminal fragments remaining intracellular. The extracellular location of the C-terminus suggests that the C-terminal fragment most likely displays a multi-transmembrane topology with additional parts being exposed extracellularly (Fig. 2A). The fate of the remaining N-terminal part of MPXV197 is currently unknown and additional work will be required to delineate the topology of this protein in more detail.

**Figure 3:**
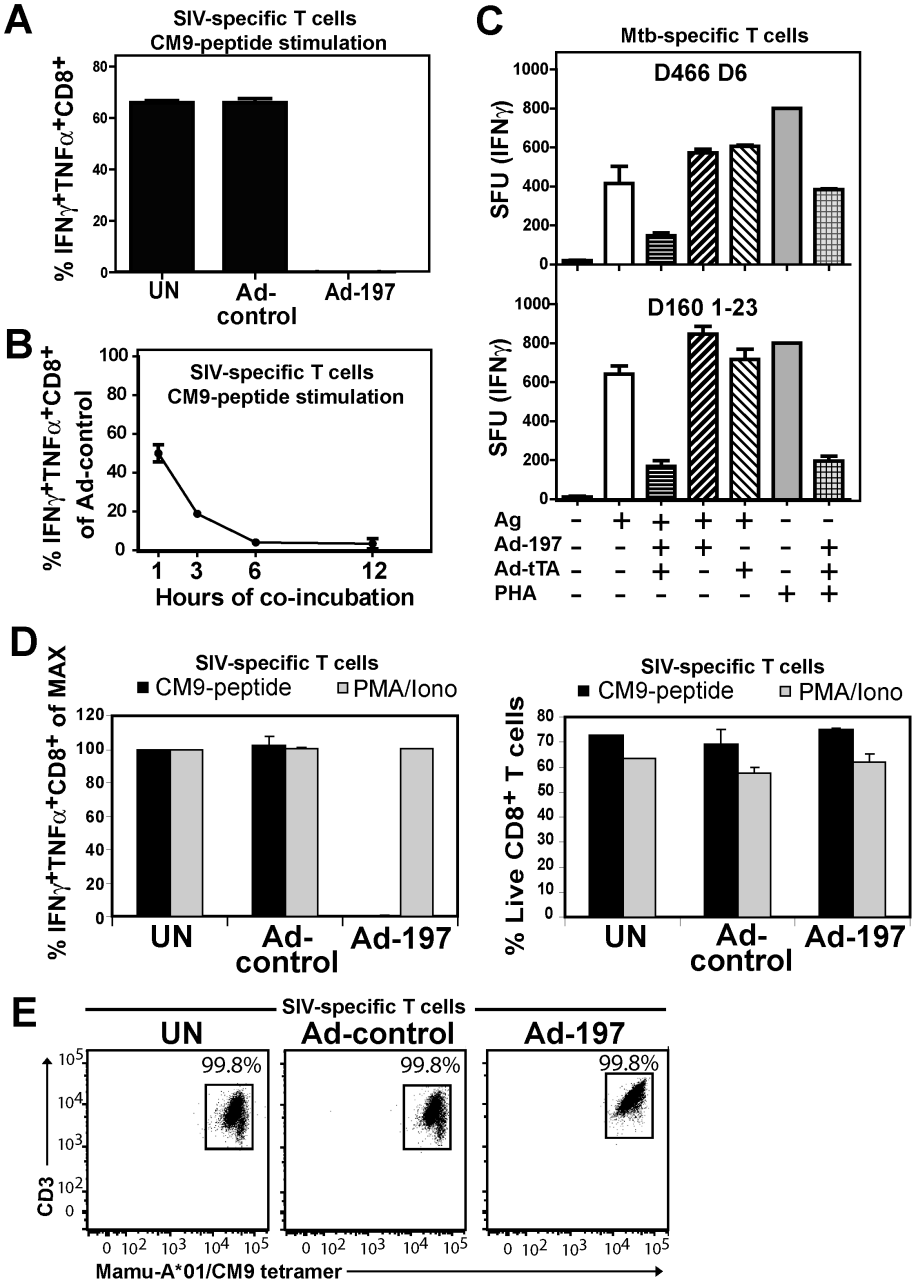
MPXV197 inhibits TCR-dependent T cell stimulation. **A)** CM9-specific CD8+ T-cells were incubated (18 h) with untreated CHO cells (UN) or CHO cells transduced with either Ad-197/Ad-tTA (‘Ad-197’) or Ad-tTA only (‘Ad-control’) and stimulated with CM9-peptide pulsed BLCLs. The percentage of INFγ+ TNFα+ CD8+ T-cells was determined by ICCS. **B)** To determine the kinetics of T cell inhibition by MPXV197 CM9-specific T-cells were incubated with Ad-197/Ad-tTA or Ad-tTA-transduced CHO cells for indicated time periods, washed, and stimulated with peptide pulsed BLCLs. **C)** Human *Mtb* specific CD8+ T cell clones D466 D6 and D160 1–23 were stimulated with BEAS-2b cells transduced with Ad-197/Ad-tTA or Ad-tTA only in the presence of CFP10_2-12_ peptide or pronase digested *Mtb* cell wall, respectively. For MHC-independent stimulation, both clones were incubated with PHA. The number of IFNγ+ T cells was measured by ELISPOT. **D)** CM9-specific CD8+ T cells were incubated (18 h) with CHO cells transduced with Ad-197/Ad-tTA or Ad-tTA, washed, and stimulated either with PMA/Iono or CM9-peptide pulsed BLCLs. Left panel: The percentages of INFγ+ TNFα+ T-cells were determined by ICCS with stimulation in the presence of uninfected CHO cells set to 100% (MAX). Right Panel: The percent live CD8+ T cells was determined by LIVE/DEAD Fixable Dead Cell Stain. **E)** MaMu-A*01/CM9 tetramer staining of CM9-pecific CD8+ T cells after 18 h of incubation with MPXV197-expressing CHO cells (‘Ad-197’) or control cells (‘Ad-control’).

To test whether MPXV197 inhibits T cell stimulation we co-incubated Ad-197 -transduced CHO cells with CM9-specific CD8+ T cells stimulated with peptide-pulsed BLCL. Thus, T cells were stimulated by exposure to cognate peptides presented by BLCLs whereas MPXV197 is provided in trans by expression in CHO cells. Since MPXV197 is under control of the tetracycline-regulated transactivator (tTA) CHO cells were co-transduced with Ad-tTA. CHO cells transduced with Ad-tTA alone did not inhibit T cell activation with cognate antigen (Ad-control, Fig. 3A). In contrast, T cell responses were reduced to ∼0.01% of control upon MPXV197 expression (Ad-197, Fig. 3A). Thus, MPXV197 inhibits T cell stimulation *in trans* even when provided by unrelated cells of a different species. To measure the kinetics of the CM9-specific CD8+ T cell inactivation we co-incubated CM9-specific T cells with MPXV197-expressing cells for variable time periods prior to stimulation with peptide pulsed BLCLs. T cell stimulation was reduced following as little as 1h of exposure to MPXV197, with maximal inhibition at 6 h of co-incubation (Fig. 3B).

Next we examined whether MPXV197 inhibits human T cell clones restricted by either classical (HLA-B) or non-classical (HLA-E) MHC-I using *M. tuberculosis*-specific CD8+ T cell clone D466 D6 recognizing peptide CFP2-12 presented by HLA-B [25] and D160 1-23 which is stimulated by pronase digested Mtb cell wall in the context of HLA-E [26]. BEAS-2B epithelial cells were infected with either Ad-197 alone or together with Ad-tTA followed by incubation with *Mtb*-specific CD8+ T cell clones. As shown in Fig. 3C, stimulation of both clones was inhibited by MPXV197. Since in this assay, MPXV197 is expressed in the same cells that present antigen we additionally examined antigen- and MHC-independent T cell stimulation of these T cell clones by phytohaemagglutinin (PHA), a lectin that activates the TCR non-specifically by carbohydrate cross-linking. PHA stimulation of both D466 D6 and D160 1–23 was inhibited by MPXV197. Taken together, these data demonstrate that MPXV197 recapitulates the T cell unresponsiveness mediated by MPXV for both human and non-human primate T cells regardless of the TCR stimulus.

### MPXV197 inhibits TCR-mediated stimulation upstream of PKC and does not induce cell death

Lack of cellular amine-reactive fluorescent staining (LIVE/DEAD Fixable Dead Cell Stain) indicates that T cell membranes remain intact in the presence of MPXV197 (Fig. 3D, right panel). To further determine whether T cells would be activated upon by-passing TCR stimulation we stimulated CM9-specific T cells with phorbol 12-myristate 13-acetate (PMA) which activates protein kinase C (PKC) and the Ca2+ ionophore ionomycin (Iono). Unlike peptide stimulation, MPXV197-expressing CHO cells did not inhibit T cell stimulation by PMA/Iono (Fig. 3D, left panel). Thus, T cells remain viable after exposure to MPXV197 suggesting that MPXV197 counteracts TCR-dependent signal transduction upstream of PKC. Moreover, exposure of CM9-specific CD8+ T cells to MPXV197-expressing CHO cells did not impair their ability to bind a MaMu-A*01/CM9 tetramer suggesting that MPXV 197 does not interfere with MHC-I peptide loading (Fig. 3E). We conclude that MPXV197 interference occurs after TCR engagement with peptide/MHC complexes most likely inhibiting TCR proximal signal transduction.

### T cell inactivation by VARV B22 and CPXV 219, members of OPXV B22- protein family

**Figure 4:**
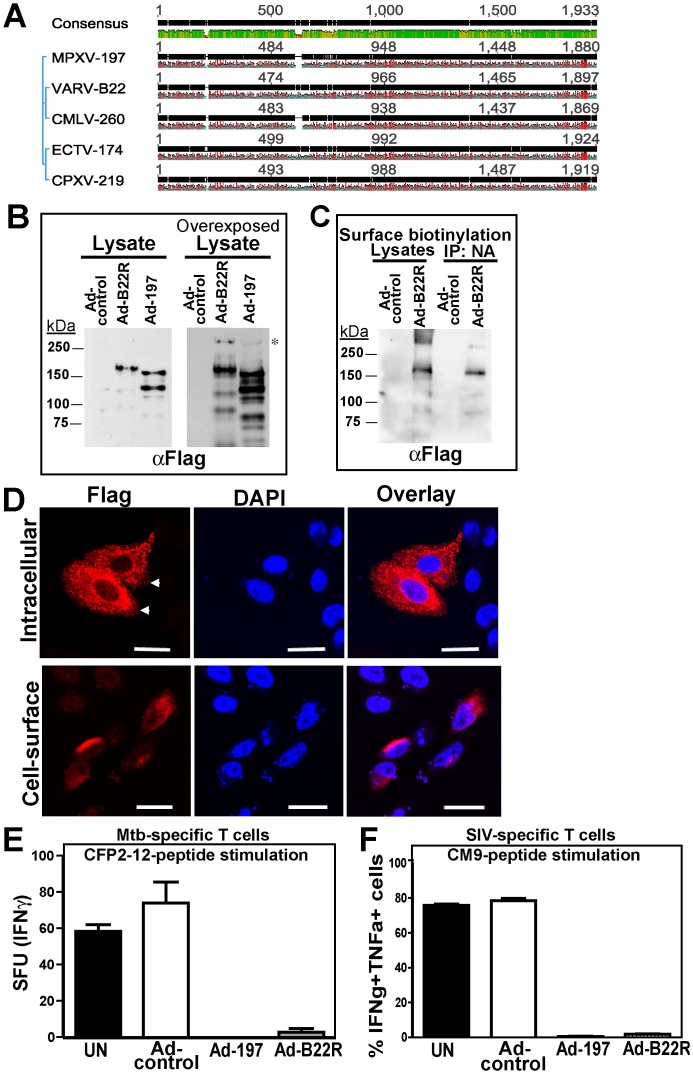
Inhibition of T cell activation by VARV B22. **A)** Sequence comparison of MPXV 197 and its orthologs by Geneious v5.6.3. Black, green, and red bars show consensus sequence, conserved, and hydrophobic residues, respectively. **B)** CHO cells were transduced with Ad-B22R, Ad-197 and Ad-tTA for 24 h followed by immunoblotting with αFLAG. Right panel: Overexposure reveals a >250 kDa band (asterisk). **C)** CHO cells were transduced as in B). After 24 h, cell surface proteins were biotinylated followed by immunoprecipitation with NeutrAvidin, electrophoretic separation and immunoblotting with αFLAG. **D)** CHO cells were transfected with pCDNA3.1-B22-CFlag (24 h), fixed, and either permeabilized (‘intracellular’) or left unpermeabilized (‘cell-surface’). The samples were stained with αFLAG and analyzed by LSCM. The scale bar is 20 µm. **E)** BEAS-2b cells, uninfected (UN) or transduced with Ad-197/Ad-tTA (‘Ad-197’) or Ad-tTA only (‘Ad-control’) were used to stimulate human *Mtb*-specific T cell clone D466 D6 with CFP10_2-12_ peptide. **F)** CM9-specific T-cells were incubated (18 h) with CHO cells either uninfected (UN) or transduced with Ad-B22R/Ad-tTA (‘Ad-B22R’) or Ad-tTA only (‘Ad-control’) followed by stimulation with CM9-peptide pulsed BLCLs.

MPXV197 belongs to the B22- protein family found in several OPXVs (Fig. 4A) including CPXV (CPXV219, 84% amino-acid identity), which causes zoonotic infections in humans, and VARV (B22, 86% amino-acid identity), the causative agent of smallpox. To examine whether VARV B22 also inhibits T cell stimulation we inserted codon-optimized B22 encoding for 1897aa with a predicted molecular mass of ∼214 kDa into expression vectors. Similar to MPXV197, immunoblots and surface biotinylation of VARV B22 revealed surface expressed ∼150 kDa fragment with the B22 fragment being slightly larger than the corresponding MPXV197 fragment (Figs. 4B,C). Also similar to MPXV197 was the observation that the full-length precursor protein was barely detectable at steady state consistent with the 150 kDa protein being the final product. The smaller protein bands were less abundant than those seen in MPXV197-expressing cells. It is possible that these minor bands in both MPXV197 and VARV B22 are by-products of high level ectopic expression. Similar to MPXV197, we observed that the C-terminus of VARV B22 is exposed at the cell surface (Fig. 4D). T cell inhibition by VARV B22 was examined using both human *Mtb*-specific CD8+ T cell clones and rhesus CM9-specific CD8+ T cell lines. As shown in Fig. 4E and F, VARV B22 inhibited T cell stimulation of both human and RM T cells as efficiently as MPXV197. These data strongly suggest that VARV inactivates T cells in a manner that is similar to MPXV.

**Figure 5:**
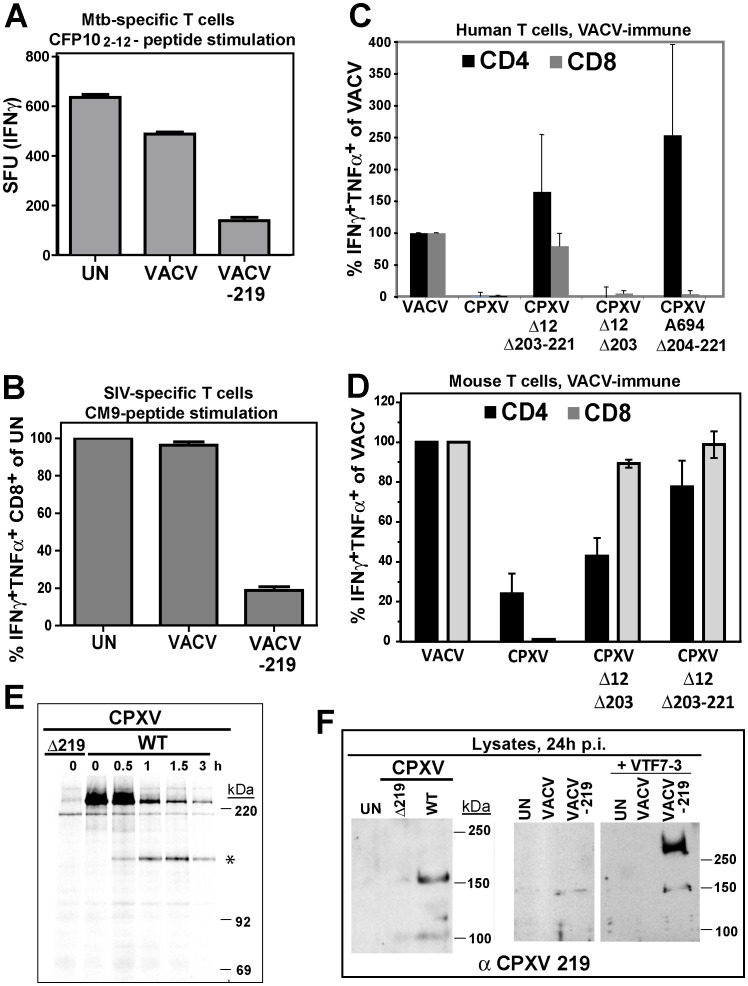
Inhibition of T cell activation by CPXV 219 is species-specific. **A)** Human *Mtb*-specific T cell clone D466 D6 was incubated with BEAS-2b cells uninfected (UN) or infected with VACV or VACV-219 (3 h) prior to addition of CFP10_2-12_ peptide. The number of IFNγ+ T cells was determined by ELISPOT. **B)** CM9-specific T-cells were incubated with HFF infected with VACV or VACV-219 for 18 h and stimulated with CM9-peptide pulsed BLCLs. The percentage of INFγ+ TNFα+ CD8+ T cells was determined by ICCS. **C)** PBMC from VACV-immune subjects (n = 3) were infected with indicated viruses (optimized MOI of 0.3-0.6) for 18 h. The infection rates for CD14+ cells were VACV (54%), CPXV (45%), CPXV Δ12Δ203-221 (51%), CPXVΔ12-203 (60%), CPXVΔ11-38 (72%), and CPXVΔ204-221 (73%). The percentage of CD4+ and CD8+ responding to poxvirus infection was determined by ICCS for IFNγ and TNFα. The frequency of VACV-reactive T cells was set to 100%. **D)** Splenocytes from VACV-immunized mice were incubated with A20 cells infected with indicated viruses (MOI 5.0) for 6 h. The frequency of poxvirus-reactive T cells was determined by ICCS for IFNγ and TNFα relative to the frequency of VACV-reactive T cells which was set to 100%. **E)** Biosynthesis of CPXV219 was studied in human 143 cells infected with CPXV wild-type or Δ219 mutant (3 h) and metabolically labeled for 45 min followed by chase for 0.5, 1, and 3 h. Cell lysates were immunoprecipitated with αCPXV219 Ab. **F)** Left Panel: Immunoblot of CHO cells infected with CPXV, CPXVΔ219 (MOI = 5.0) or uninfected (UN) using αCPXV219 Ab. Right Panel: Immunoblot with αCPXV219 Ab of CHO cells infected with VACV, VACV-219 (MOI = 5.0) or uninfected (UN), or co-infected with T7-polymerase expressing VACV VTF7-3 (MOI = 5.0).

To determine whether CPXV219 would similarly inhibit T cells we used a recombinant VACV expressing CPXV219 to infect BEAS-2B cells and examine stimulation of *Mtb*-specific T cells or to infect HFF and monitor stimulation of SIV-specific T cells by CM9 peptide loaded BCBLs. Whereas VACV did not impact stimulation of human or RM T cells, VACV-219 inhibited T cell stimulation in both instances (Fig. 5 A, B). Thus it seems that the B22 proteins represent a family of T cell inhibitors.

The finding that CPXV219 inhibits T cells was unexpected since we previously reported that poxvirus-specific T cells were stimulated once MHC-I-dependent antigen presentation by CPXV was restored due to deletion of CPXV12 and CPXV203 [20]. However, genome analysis of our deletion virus CPXVΔ12Δ203 revealed that, upon passaging, this mutant had acquired additional deletions downstream of CPXV203 due to a recombination event resulting in ORF204-221 being replaced by a duplication of ORF10-11 (data not shown). Therefore, this deletion virus (now designated CPXVΔ12Δ203-221) lacks not only CPXV12 and CPXV203, but also CPXV219. However, an independently generated CPXVΔ12Δ203 mutant was also reported to stimulate poxvirus-specific T cells [18], although this analysis was limited to murine T cells. To determine the impact of CPXV219 on human and mouse T cells we analyzed CPXV mutants lacking CPXV219 alone or together with CPXV12 and CPXV203 and mutants lacking CPXV12 and CPXV203. Stimulation of poxvirus-specific human T cells was analyzed by infecting PBMC from VACV-immune subjects with CPXV and monitoring T cell activation by ICCS whereas stimulation of murine T cells was monitored by adding splenocytes from VACV-immunized mice to CPXV-infected A20 cells (Fig. 5C,D).

CPXV did not stimulate poxvirus-specific human CD8+ and CD4+ T cells whereas mutant A694 lacking the genomic region CPXV204-221 stimulated human CD4+ T cells but not CD8+ T cells (Fig. 5C). Since A694 contains CPXV12 and CPXV203 these data suggest that human CD8+ T cells are not stimulated due to MHC-I evasion whereas human CD4+ T cells were stimulated due to the absence of CPXV219. Indeed, CPXVΔ12Δ203-221 lacking CPXV219 as well as CPXV12 and CPXV203 restored stimulation of both human CD4+ and CD8+ T cells. In contrast, CPXVΔ12Δ203 did not restore stimulation of poxvirus-specific human CD8+T cells (Fig. 5C), despite the restoration of MHC-I presentation [18]. These results are consistent with CPXV219 inhibiting both human CD4+ and CD8+ T cells in a manner similar to MPXV197. However, when stimulation of murine poxvirus-specific T cells was examined with the same series of mutants, CD8+ T cells were stimulated in the absence of CPXV12 and CPXV203 even when CPXV219 was present (Fig. 5D) as reported [18]. Interestingly however, CPXVΔ12Δ203 showed reduced activation of CD4+ T cells compared to VACV or CPXVΔ12Δ203-221 suggesting that CPXV219 does not efficiently inactivate murine CD8+ T cells but might impact murine CD4+ T cells. Together with the finding that MPXV stimulated murine CD8+ T cells (data not shown) these results indicate that B22 proteins inhibit human and monkey T cells, but are less active against murine T cells.

Using a rabbit anti-serum raised against purified GST-tagged CPXV219 we examined its expression in CPXV-infected human 143 cells and CHO cells as well as in HEK 293 cells infected with VACV-219 (Figs. 5E,F). CPXV219 was expressed with early kinetics and detectable as early as 3h p.i. (data not shown). Metabolic pulse/chase labeling and immunoprecipitation at 3 h p.i. further demonstrated that a high molecular mass product (>220 kDa) was processed into a ∼150 kDa fragment (Fig. 5E). Consistent with the ∼150 kDa fragment being the final product, a similarly sized protein was the predominant fragment in immunoblots of CPXV-infected CHO cells whereas this was absent from CPXVΔ219-infected cell lysates (Fig. 5F). Similarly, a ∼150 kDa fragment was the predominant protein found in VAC-219 infected cells in the absence of the T7 polymerase. However, upon co-infection with T7-polymerase expressing VACV, the >250 kDa precursor was highly expressed whereas the ∼150 kDa fragment was only slightly increased consistent with the majority of the protein remaining in the ER-resident precursor state upon overexpression. Taken together with the data shown above for MPXV197 and VARV B22, these data suggest that in both virally infected and ectopically expressing cells the full-length precursor protein is processed into a ∼150 kDa fragment. Since the anti-CPXV219 antiserum was raised against the whole protein, it is not known which part of the protein is recognized. However, the ∼150 kDa fragment of both MPXV197 and VARV B22 was detected by a C-terminal FLAG-tag suggesting that the CPXV219 ∼150 kDa fragment is likewise C-terminal. Thus, we conclude that a ∼150 kDa C-terminal fragment is the ultimate product of MPXV197, VARV B22 and CPXV219 and that this fragment is transported to the cell surface where it acts as T cell inactivator.

### MPXVΔ197 is attenuated *in vivo*

Since B22 proteins are more active against primate than rodent T cells we used a recently described intrabronchial (i.b.) inoculation model in RM [27] to determine the role of MPXV197 in viral dissemination, pathogenesis and induction of T cell responses. To rule out that MPXVΔ197 contained additional mutations compared to parental strain MPXV-US2003 we sequenced the genomes for both viruses by next generation (NextGen) sequencing. Within a margin of error (<3%) both WT and MPXVΔ197 matched the predicted sequence exactly (Fig. S3, Table S2). Since this analysis cannot distinguish between sequencing errors, misalignments (particularly in the repeat region) and actual mutations, it is likely that the actual percentage of correct genome sequences is substantially higher. Therefore, we conclude that the vast majority of genomes present in our WT control and MPXV197-deleted virus contain the expected genome sequence.

**Figure 6:**
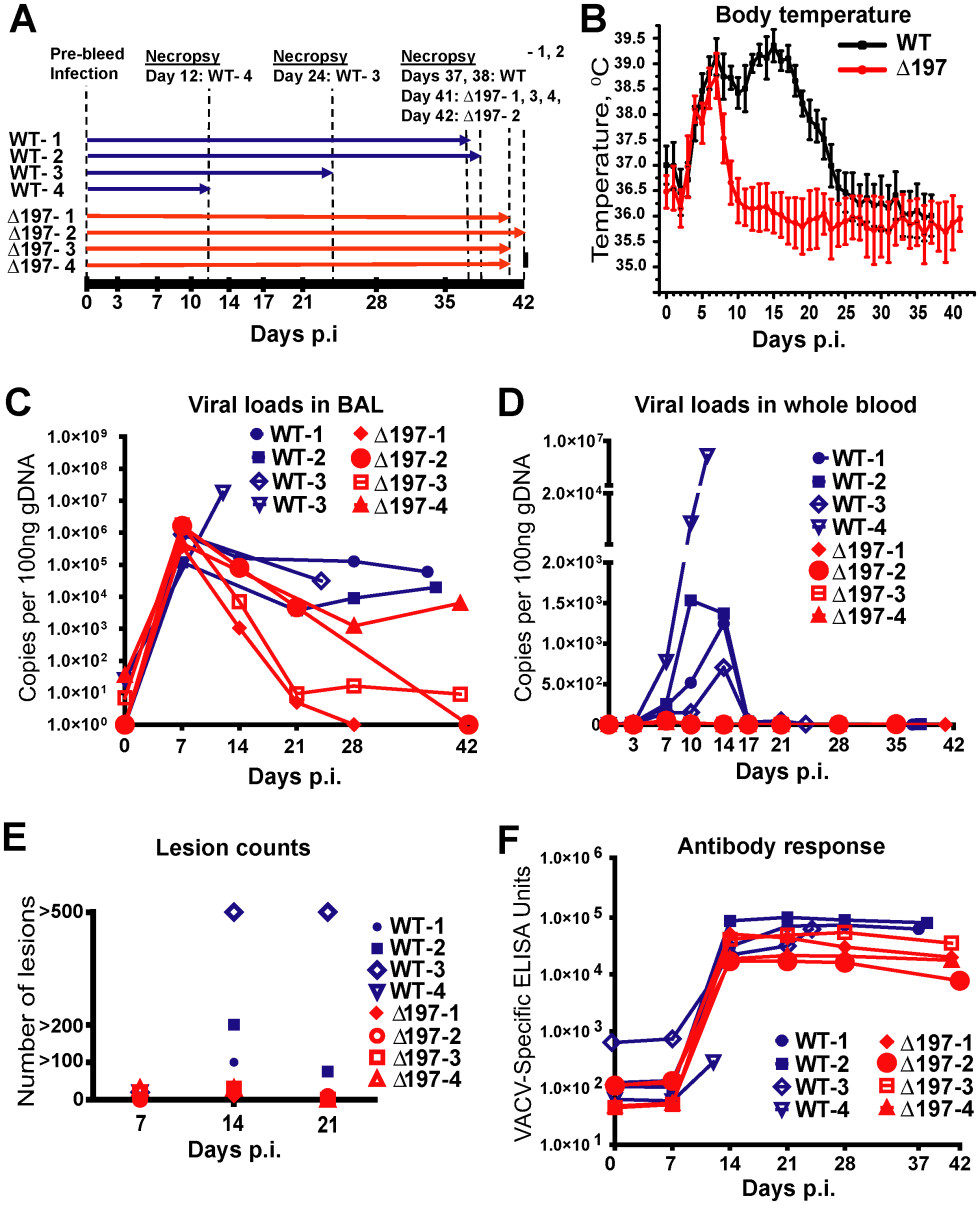
MPXVΔ197 is attenuated *in vivo*. **A)** 4 female RM were inoculated i.b. with 2×10^5^ PFU of MPXV US2003 (WT) or MPXVΔ197 on day 0. Whole blood, BAL, and PBMC samples were taken on indicated dpi. 2 RM infected with MPXVUS2003, WT-4 and WT-3, were euthanized at 12 and 24 dpi, respectively. The remaining WT-infected were euthanized on days 37 and 38 pi. Animals infected with MPXVΔ197 were euthanized at 41 and 42 dpi. **B)** Average nighttime body temperature (7PM to 7AM) as determined by biotelemetry transmitters for RM infected with WT (black) or MPXVΔ197 (red) (mean +/− SEM). P = 0.0007 (area under curve (AUC), F-test). **C)** and **D)** Viral loads determined by qPCR in BAL (**C**) and whole blood (**D**) of WT (blue) or MPXVΔ197 (red)-infected RM. P = 0.003 (AUC, F-test) and P<0.0001 (AUC, F-test) for BAL and whole blood, respectively. **E)** Number of skin lesions in WT (blue) or MPXVΔ197 (red)-infected RM. The *p*-value for the AUC comparison is P = 0.0003 (F-test). **F)** Poxvirus-specific antibody titers were determined by ELISA using VACV as antigen. The titers were not statistically different between WT and MPXVΔ197 cohorts.

We infected 8 RM with MPXV-US2003 or MPXVΔ197 using i.b. inoculation of 2×10^5^ PFU (Fig. 6A), a dose at which MPXV-Zaire was non-lethal [27]. The clinicopathologic course of infection was followed by physical examination, biotelemetry to record body temperature and activity, O_2_ tissue saturation, and development of cutaneous lesions. Blood and bronchoalveolar lavage (BAL) fluid samples were collected at defined days post infection (dpi) to determine the kinetics of virus replication and of the adaptive immune response. As shown in Fig. 6 and table S3, RM infected with MPXVΔ197 experienced a significantly shorter duration of fever (5 days compared to 20 days) (Fig. 6B), fewer skin lesions (Fig. 6E), and dramatically reduced morbidity and mortality. In fact, two of the MPXV-US2003-infected RM had to be euthanized due to deteriorating health whereas all four of the MPXVΔ197-infected RM spontaneously controlled the infection prior to termination of the experiment at days 41 and 42. Viral titers measured in the lungs were initially similar, reflecting the similar size of the inoculum, but lung titers of MPXVΔ197 fell significantly more rapidly compared to WT (Fig. 6C). An even more striking contrast was observed for viral titers in the blood where all RM infected with MPXV-US2003 showed significantly higher levels of viremia compared to MPXVΔ197 which was barely detectable (Fig. 6D). Interestingly, while uncontrolled viremia in both lungs and blood correlated with rapid deterioration of health in one animal (WT-4), the other animal that needed to be euthanized prematurely (WT-3) had a lower viremia in the blood but a higher number of lesions at days 14 and 21 compared to the remaining WT-infected RM (Fig. 6E). In contrast, low titers in the blood correlated with a generally mild disease and less than 30 lesions in MPXVΔ197-infected RM (Fig. 6E, Table S3). Decreased viral titers of MPXVΔ197 were also reflected in a decrease of antibody titers which tended to be lower than that of MPXV-US2003 although this was not statistically significant (Fig. 6F).

**Figure 7:**
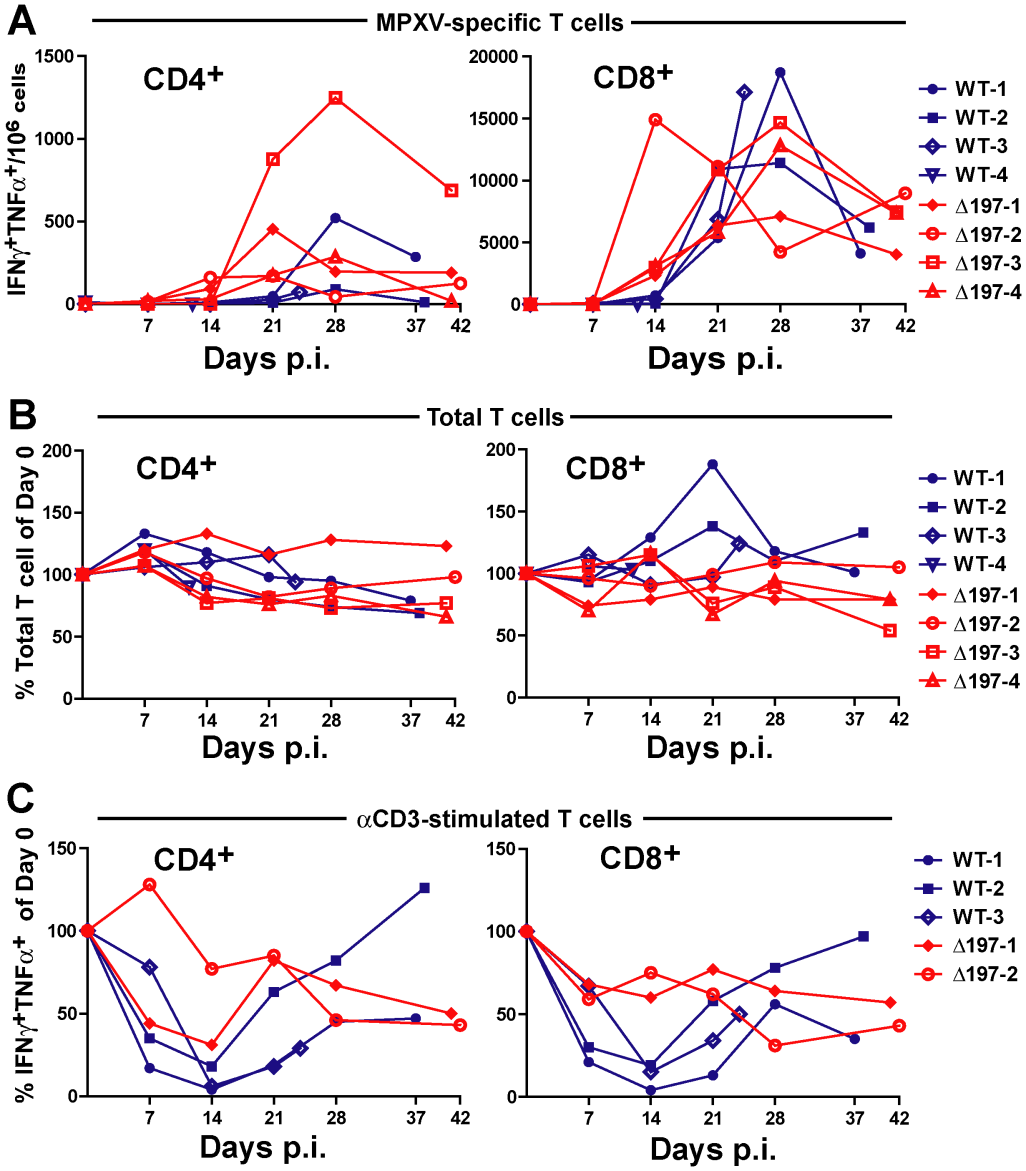
MPXV197 suppresses T cell responses *in vivo*. **A)** PBMC from WT (blue) and MPXVΔ197 (red)-infected RM were infected with VACV (MOI of 0.3) for 18 h. The background-subtracted frequency of poxvirus-responsive CD4+ and CD8+ T cells was determined by ICCS for TNFα and IFNγ. The differences were statistically significant at day 21 (P = 0.0063, F-test) for CD4+ T cells and at day 14 (P = 0.0069, F-test) for CD8+ T cells. **B)** The total frequency of CD4+ and CD8+ relative to day 0 as determined by flow cytometry is shown. The frequencies were not statistically different between WT and MPXVΔ197 cohorts. **C)** The percentage of CD4+ and CD8+ T cells relative to day 0 responding to anti-CD3 stimulation was determined by ICCS for IFNγ and TNFα. PBMC from WT (blue) or MPXVΔ197 (red) infected animals were stimulated with plate-bound αCD3 Ab for 6 h. The differences were statistically significant at day 14 (P = 0.0065, two-tailed t-test) for CD8+ T cells.

In stark contrast to the reduced virologic and disease parameters, poxvirus-specific T cell responses were detected earlier and were significantly higher at some of the earliest time points in RM infected with MPXVΔ197 compared to MPXV-US2003 (Fig. 7A). (Note that T cell responses were measured using VACV to avoid the T cell inhibitory effect of MPXV197). At day 14, all four MPXVΔ197-infected RM had a significantly higher frequency of poxvirus-specific CD8+ T cells in their blood compared to the 3 remaining WT-infected RM (Fig. 7A). Similarly, in 3 of 4 MPXVΔ197-infected RM the CD4+ T cell response was above background at days 7 and 14 whereas 0/4 or 2/3 WT-infected RM had detectable CD4+ and CD8+ T cells at these days. At day 21, the frequency of CD4+ T cells in all MPXVΔ197-infected RM was significantly higher than in WT-infected RM. The inverse correlation between viral titers and T cell responses in the blood is consistent with MPXV197 contributing to viral dissemination during the early phase of infection by delaying the onset of the cellular immune response.

To examine whether the T cell inactivation mediated by MPXV197 would result in a systemic suppression of T cell responses during viral infection *in vivo*, we stimulated T cells in PBMC with αCD3 Ab. The data is limited to three WT and two MPXVΔ197-infected RM since two animals were missing samples and T cells from Δ197-3 was unresponsive to αCD3 stimulation potentially due to CD3 polymorphism. Although the overall frequency of T cells in the blood did not change during infection (Fig. 7B), there was a dramatic reduction in αCD3 responses of both CD4+ and CD8+ T cells from WT-infected RM at 7–21 dpi (Fig. 7C). This was particularly evident at day 14 which correlated with peak viremia in the blood of WT-1 and WT-2-infected animals (Fig. 6D). In contrast, this decrease was less pronounced for αCD3-stimulation of T cells in both MPXVΔ197-infected RM. Although not statistically significant due to the low sample size, these observations are consistent with MPXV197 contributing to a systemic suppression of T cell responses during peak viremia.

## Discussion

We report here a novel mechanism by which viral proteins inhibit T cell responses and describe the impact of this immunomodulation on viral virulence and immunity. The activation of T cells via TCR engagement with peptide/MHC complexes is the principal mechanism by which CD8+ T cells recognize and eliminate virus-infected cells and by which CD4+ T cells recognize APC. To limit T cell control, viruses can thus either interfere with antigen presentation or they can interfere with the ability of T cells to respond to antigen. Many instances of the former mechanism have been described, particularly for large DNA viruses that are limited in their ability to escape from T cell control by mutating immunodominant epitopes [28]. However, MHC-inhibitors are unable to prevent activation of T cells by non-infected APC processing exogenous antigens via MHC-II presentation or by MHC-I cross-presentation. In contrast, B22 proteins would allow poxviruses to interfere with TCR-dependent T cell activation regardless of the stimulatory pathway.

While indirect inhibition of T cell stimulation, e.g. by interference with cytokine networks [29], [30], [31], [32] has been described for poxviruses, very few instances of viruses directly inhibiting T cells have been reported to date. Among poxviruses, a secreted CD30 homologue of ECTV was shown to inhibit CD4+ T cells activation in mixed lymphocyte reactions [33]. However, the CD30 homologue encoded by the CPXV strain used in our studies did not seem to affect T cell stimulation since both human and mouse T cells were activated upon elimination of the MHC-I inhibitory genes CPXV12 and CPXV203 and the T cell inactivator CPXV219. It was also reported that VACV inhibits TCR-dependent responses of γδ T cells [34], but this inhibitory mechanism clearly does not apply to αβ T cells since we used VACV as our control in all T cell experiments. To our knowledge, the most closely related mechanism described so far for a viral protein interfering with T cell stimulation was reported for UL11 of human cytomegalovirus (HCMV) which binds to CD45 on the surface of T cells and inhibits TCR-mediated signaling [35]. However, UL11 is unable to prevent the stimulation of HCMV-specific T cells by fibroblasts infected with HCMV lacking viral MHC-I inhibitors [36]. In contrast, deletion of CPXV MHC-I inhibitors did not restore human T cell stimulation when CPXV219 was present (Fig. 5C). Moreover, MPXV seems to rely solely on MPXV197 for T cell inhibition. Thus, the strong inhibitory effect of B22 proteins stands out since it seems to require only a brief interaction with T cells, even non-cognate T cells, to shut down T cell stimulation. Since effector T cells are exquisitely sensitive to activating signals, one peptide/MHC complex reportedly can activate cytokine secretion in T cells [37], the T cell inhibition by B22 family proteins is truly remarkable. This silencing of T cells is likely transient since T cells respond to stimuli that by-pass TCR signaling such as PMA/Ionomycin in the presence of MPXV197 (Fig. 3D). The B22 inhibitory impact could thus be best described as transient inactivation, or transient “functional paralysis”, a term previously suggested for HCMV UL11 [35].

The molecular mechanism by which B22 proteins inactivate T cells is currently unknown. A multitude of proteins expressed on T cells can exert negative signals, in many cases overriding the ability of the TCR to signal. Engagement of these inhibitory receptors results in peripheral T cell tolerance that can be either permanent, as in T cell exhaustion, or temporary. Therefore, these proteins are often referred to as immune checkpoint proteins or co-inhibitory proteins [38]. Conceivably, B22 proteins could exert their effect by engaging one or several of these co-inhibitory proteins. Since B22 proteins seem to be unable to block murine CD8+ T cells it seems that the target protein on T cells is either primate-specific or that it is differentially expressed on T cells in mice. The inability of these proteins to inhibit murine T cells might be the reason why this effect has not been observed previously when studying CPXV in mice. Since ECTV is a mouse pathogen it will be interesting to examine whether the ECTV B22 homologue is capable of inhibiting mouse T cells. If not, the question arises whether B22 proteins perform a different function in rodents, or whether the lack of T cell inhibition is only observed in the genus *mus*, but not in other rodent species. Since both CPXV and MPXV infect rodents in the wild, it would be interesting to explore the species-specificity of this protein family in more detail. Additionally, B22 proteins might perform different functions in different species. For instance it is conceivable that in addition to the membrane bound ∼150 kDa C-terminal fragment, a secreted amino-terminal fragment is generated that has functions other than T cell inactivation.

In contrast to MPXV and CPXV which display a rather wide host range, VARV is highly restricted to humans which enabled the eradication of this virus. The finding that VARV B22 inhibits human T cells, together with the finding that the corresponding protein in MPXV contributes dramatically to virulence, suggests that the presence of B22 might have contributed to the devastating pathology of smallpox infection. It is estimated that smallpox caused as many as 300–500 Million deaths in 20^th^ century alone [39] rendering this virus one of the most, if not the most, virulent infectious disease ever to have affected the human population. Our data suggest that T cell evasion by B22 potentially contributed to this deadly outcome.

MPXV197 is the first MPXV protein that was shown to have such a dramatic impact on virulence. Previous observations demonstrated that deletion of a viral complement modulator from MPXV-Zaire slightly increased the virulence in RM [27]. Of note, the mutant virus used in this previous experiment contained the same GFP/GPT expression cassette as the one used to replace MPXV197 thus eliminating the possibility that the attenuation of MPXVΔ197 was due to the presence of these heterologous gene product which were absent from the control virus. Interestingly, the complement binding protein is absent from West-African strains of MPXV including MPXV-US2003 used in our experiments as WT virus. The West-African clade is assumed to be less virulent than the central African clade in the human population [2] and the US2003 strain has been shown to be less virulent in small animal models of MPXV [3], [4], [5] as well as in a small study in cynomolgus macaques [6]. In our experiment, however, the US2003 strain was highly virulent since two of the four monkeys had to be euthanized when given a dose of 2×10^5^ PFU. This mortality is similar, if not higher, than the mortality of RM infected with MPXV-Zaire at the same dose [[27] and unpublished observations].

Compared to the high morbidity and mortality observed with MPXV-US2003, infection with MPXVΔ197 resulted in a comparatively benign disease that was controlled by the host. Viral load at the primary site of infection in the lungs was initially similar to WT, indicating that initial viral replication does not depend on MPXV197 consistent with MPXV197- deletion having no effect on viral replication *in vitro* (Fig. S2B). In contrast, MPXV197 is required for efficient dissemination since both blood titers and skin lesions were reduced by orders of magnitude compared to WT. This effect correlates both with the timing and role of T cell responses in limiting poxviral disease. In WT-infected RM, T cell responses developed in 3 weeks and peaked around 4 weeks pi. In those animals that survived the infection with WT-virus, the development of measurable T cell responses coincided with control of viremia and reduction in fever that occurred between 2 and 3 weeks pi. By comparison, T cell responses to MPXVΔ197 were already detectable at 2 weeks pi and peaked around 3 weeks. In all MPXVΔ197-infected RM had returned to normal and viremia was no longer detectable by 10 dpi. The T cell response to MPXVΔ197 is thus similar to that observed for VACV which is clearly detectable at 2 weeks pi [16]. In contrast, the delayed T cell response to WT MPXV is likely mediated by MPXV197 which presumably contributes to the significant virulence of MPXV in the human host. The reduced spreading from lungs to blood is thus most likely an indirect effect of T cells more efficiently controlling the dissemination of MPXVΔ197 compared to MPXV US2003.

Previously it was shown that deletion of the MHC-I downregulating molecules CPXV12 and CPXV203 decreased CPXV mortality in mice [18] and deletion of MYX153 decreased virulence of myxoma virus in rabbits [40]. Taken together with our observations, these data suggest that the ability to limit T cell responses increases poxviral virulence in a number of poxvirus species.

Our data also suggest that MPXV197 contributes to a temporary systemic immune suppression during acute infection. Systemic T cell suppression *in vivo* by an OPXV protein has not been previously observed. In contrast, generalized immune suppression mechanisms that involve peripheral T cell dysfunction are well known for chronic infection with viruses such as HIV, HBV, HCV, or chronic strains of LCMV [41]. However, T cell dysfunction in these cases is not the result of T cell shut-off by dedicated viral proteins but rather involves viral interference with immune regulatory networks, such as T cell exhaustion due to persistent antigen exposure or depletion of dendritic cells by viral infection [41]. Thus, systemic immune suppression by dedicated viral proteins inhibiting TCR signal transduction could represent a novel mechanism of viral T cell dysregulation. Such systemic T cell inactivation could also impact the priming of T cells since it would broadly inactivate the ability of T cells to respond to TCR-stimulation. T cell priming could either be delayed or the specificity of primed T cells could be skewed towards epitopes that are less affected by T cell inactivation, e.g. cross-presented epitopes versus epitopes presented by infected cells.

The efficiency with which B22 proteins shut down T cells suggests that it might be possible to use such proteins to therapeutically reduce unwanted T cell-mediated inflammation. Using poxviral immunomodulators to control unwanted immune responses has already been demonstrated in a recent phase II clinical trial in which an anti-inflammatory poxviral protein of the Serpin-family significantly reduced myocardial damage biomarkers in patients receiving percutaneous coronary interventions such as coronary angioplasty or stent implantation [42]. This work establishes a proof-of-principle for the therapeutic use of viral immune modulators. Alternatively, B22 proteins might reveal novel inhibitory proteins on T cells or novel insights into T cell regulation that can be targeted to counter unwanted T cell responses. The precise mechanism of T cell inactivation by B22 proteins is therefore expected to provide novel insights into mechanisms of peripheral tolerance.

## Materials and Methods

### Cells and viruses

Human foreskin fibroblasts (HFF), BEAS-2B human bronchial epithelium cells, human 143 cells, Chinese hamster ovary (CHO) cells, and human embryonic kidney (HEK) 293 cells were maintained in Dulbecco's modified Eagle's medium (DMEM, Mediatech, Manassas, VA) supplemented with 10% fetal bovine serum (FBS, Hyclone Laboratories, Inc, Logan, UT). Rhesus macaque (RM) B-lymphoblastoid cell line (BLCL) was grown in 10% FBS-RPMI 1640 medium (Hyclone Laboratories, Inc). Mtb-specific T cell clones and monkey CM9-peptide specific T cell lines and were maintained as previously described [23], [25], [26]. BSC40, African Green Monkey kidney cells were grown in minimum essential medium (MEM, Mediatech). Jurkat T cells clone JJK were grown in 10% FBS-RPMI 1640 medium (Hyclone Laboratories, Inc).

Vaccinia virus (VACV) Western Reserve strain, monkeypox virus (MPXV) strains Zaire and US2003, Cowpox virus (CPXV) Brighton Red strain were propagated in BSC40 cells maintained in 5% FBS MEM. The virus preparations were purified using standard protocol [43] with minor modifications. Briefly, the infected cells were harvested, resuspended in 10 mM Tris-HCl (pH 8.0), and lysed by three cycles of freezing-thawing followed by two cycles of sonication. Precleared cell lysate was layered onto 36% sucrose cushion and centrifuged at 40,000×g for 80 min. Pelleted virus particles were resuspended in 1 mM Tris-HCl (pH 8.0) and titered. For complete genome sequencing and in-vivo studies, the virus was additionally purified by centrifugation (22,500×g, 40 min) through a 25% to 40% continuous sucrose gradient.

### Human subjects

VACV-immune subjects provided informed written consent before signing research authorization forms that complied with the US Health Insurance Portability and Accountability Act (HIPAA) in addition to a medical history questionnaire. These studies were approved by the Institutional Review Board of OHSU.

### Animals

All animal studies were carried out in strict accordance with the recommendations in the Guide for the Care and Use of Laboratory Animals (8th edition, The National Academies Press) and the Animal Welfare Act (the National Institutes of Health Office of Laboratory Animal Welfare assurance number A3304-01). All animal procedures were performed according to protocols #0865 and #0731 approved by the Institutional Animal Care and Use Committee of the Oregon Health and Science University. Appropriate sedatives, anesthetics and analgesics were used during handling, and clinical and surgical procedures to ensure minimal pain, suffering and distress to animals.

Female BALB/c mice at 5 months of age were purchased from The Jackson Laboratory. Mice were immunized intraperitoneally (i.p.) with 2×10^6^ PFU/mouse of VACV WR. On day 8 post inoculation, spleens were collected and used for studies of the T cell responses to CPXV BR wild type and mutants.

Eight adult female RM animals were utilized for *in-vivo* studies of the T cell responses to MPXV US2003 wild type (WT) and MPXVΔ197 mutant. Cohort 1 (WT) included animals 29437 (WT-1; 7 year-old), 29785 (WT-2; 10-year-old), 21111 (WT-3; 13-year-old), and 28689 (WT-4; 13-year-old). Cohort 2 (Δ197) included animals 29792 (Δ197-1; 8-year-old), 29398 (Δ197-2; 11-year-old), 29424 (Δ197-3; 13-year-old), 28664 (Δ197-4; 10-year-old). The animals were infected intrabronchially with 5×10^5^ PFU/animal of WT and the mutant viruses delivered in 1 ml of phosphate-buffered saline (PBS). Blood and bronchoalveolar lavage (BAL) samples were collected on the day of infection (day 0) and later on indicated days p.i (Fig. 7A). Peripheral blood mononuclear cells (PBMC) were isolated from blood by centrifugation over Lymphocyte Separation Media. Body temperature and physical activity were monitored via telemetry implants (Mini Mitter, Bend, OR).

### Construction of expression plasmids

Codon-optimized sequences of the C-terminal 3×FLAG (DYKDHDGDYKDHDIDYKDDDDK) fusions of MPXV197 and VARV B22 proteins were synthesized at GenScript (Piscataway, NJ). MPXV197 N-terminal Flag fusion was constructed by removing 3×FLAG sequence from the C-terminus of the protein and inserting it downstream of the predicted signal sequence after the amino acid E_21_. All coding sequences were cloned into pCDNA3.1 vector (Life Technologies). Additionally, MPXV 197-CFlag and VACV B22-CFlag coding sequences were sub-cloned in pAdtet7 shuttle vector [44] under a tetracycline (tet) regulated promoter. The resulting plasmids were used for construction of the recombinant adenoviruses viruses. To achieve the protein expression these viruses were co-infected with Ad-tTA virus expressing tet-transactivator (tTA) protein [45]. *In vitro* synthesis of VARV B22R ORF and all *in vitro* experiments using B22R-expressing constructs were approved by the World Health Organization (WHO).

### Recombinant viruses

#### Recombinant MPXV

All work with this virus and the recombinant derivative was conducted in accordance with institutional guidelines for biosafety at OHSU. MPXV deletion mutants in US2003 strain were generated via homologous *in vivo* recombination [46] replacing up to ∼10 kb fragments with a GFP-GPT cassette. Recombination plasmids were constructed by splicing regions upstream and downstream of the indicated ORFs to the 5′ and 3′ termini of the cassette expressing green-fluorescent protein (GFP) and guanine-hypoxanthine phosphoribosyltransferase (GPT) using spice overlap extension by PCR technique [47]. The nucleotide sequences were PCR-amplified from MPXV US2003 genomic DNA and pT7 E/L EGFP-GPT vector [48] kindly provided by Dr. G. McFadden (University of Florida, Gainesville, FL) and subsequently fused by PCR using primers described in Supplemental Table 1. The resultant fragments were cloned into pCR2.1-TOPO-TA vector (Life Technologies, Grand Island, NY) using the manufacturer's protocol.

For *in vivo* recombination, BSC40 cells transfected with a recombination plasmid were infected with MPXV US2003 at a multiplicity of infection (MOI) of 0.1 and incubated for 48 h. Recovered virus was passaged twice in GPT selection medium, 5% FBS MEM – 32 g mycophenolic acid/ml – 250 µg xanthine/ml – 15 µg hypoxanthine/ml and then plaque purified. The resultant recombinant viruses were amplified and purified by centrifugation through sucrose. To verify the deletion and the absence of contaminating wild-type virus, viral genomic DNA was purified with DNeasy kit (QIAGEN, Valencia, CA) and tested by PCR using primers specific to the flanking regions. Additionally, to confirm that no other major deletions or mutations were acquired during construction of Δ197 mutant, genomic DNA of both the wild type and the mutant viruses was sequenced by using complete genome sequencing.

Recombinant MPXVΔORF184 deletion mutant in Zaire strain was generated by replacing the CPXV203 orthologue ORF 184 with a GFP-GPT cassette using the same protocol

#### Recombinant CPXV

CPXV Δ12Δ203-221 is a spontaneously derived mutant from previously described CPXV Δ12Δ203 recombinant virus [20]. Initially ORFs 12 and 203 were replaced with (E/L Pr.)GFP-(7.5 K Pr.)GPT and (7.5 K Pr.)Neo-(p4b Pr.)RFP expressing cassettes, respectively. Upon passaging, ORFs 204–221 were replaced with an inverted copy of ORFs 10–11, likely due to homologous recombination between the inverted copies of vaccinia 7.5 K promoter driving expression of both GPT and Neo and the inverted terminal repeats of the viral genome. The duplication of terminal ORFs was confirmed by PCR and sequencing of genomic DNA.

CPXVΔ12 Δ203, a mutant virus with deleted ORFs 12 and 203 and CPXV Δ203 in which ORF 203 was replaced with a GFP-expressing cassette were obtained from Dr. Wayne M. Yokoyama and were previously described [18], [19].

CPXV Δ204–221 (A694) is a spontaneously generated white pock variant (W3 variant) of CPXV (Brighton red strain) isolated and initially described by [49]. Subsequent sequence analysis showed that in comparison to the genome of the wild-type virus, the genome of this variant has lost the 33.7 kb region from nucleotide 190,832 to the right-hand end of the genome (nucleotide 224,499), with the deleted region replaced by an inverted copy of the left-hand end of the genome encompassing nucleotides 1–15,461.

CPXVΔ219 (A618) mutant that lacks 96% of the 5759-nucleotide coding region of CPXV219 was constructed via homologous recombination *in-vivo* as described above. Plasmid p1889 was generated containing GPT gene under the control of the vaccinia virus p7.5 promoter flanked by *Xma*I sites within a pGem7zf vector as described [50]. The *gpt* gene was then flanked by the XhoI- MfeI fragment (residues 205202–205719) and the HinPI-ClaI fragment (residues 209953–211788) at the 5′ and 3′ ends of the CPXV219 gene, to create plasmid p1903, which was used to create the mutant virus.

#### Recombinant VACV

VACV-219 corresponds to recombinant VACV A625 that expresses the CPXV219 gene under the control of the bacteriophage T7 RNA polymerase. The CPXV219 coding region was placed into the insertion vector pTM1 [51] by first inserting PCR products of the 5' and 3' ends of the CPXV219 coding region such that the initiation codon was at the NcoI site in pTM1, an XhoI site was downstream of the stop codon, and unique restriction sites SphI and BssHI present at the two ends of the coding region were present in the modified pTM1 plasmid. The PCR modifications were done using primers NcoI-219-5′-SphI-F and NcoI-219-5′-SphI-R (Table S1) to produce the 5' end fragment containing the SphI site, with primers BssH1- 219-3′- XhoI-F and BssH1- 219-3′- XhoI-R (Table S1) to produce the 3' end fragment containing the unique BssHI site. Then the 5526 kbp SphI-BssH1 fragment of cloned CPXV DNA in plasmid p1906 containing the entire CPXV219 gene was inserted into the modified pTM1 vector to create plasmid p1951 in which the full-length CPXV219 gene is under the control of the T7 promoter. This plasmid was used to create a recombinant VACV-219 via homologous recombination *in-vivo* as described [52]. The expression of CPXV219 in cells co-infected with VACV-219 and VTF7-3 [53], a VACV expressing the phage T7 polymerase, was confirmed by immunoprecipitation of proteins metabolically labeled with [35S] methionine and immunoblot (Fig. 5F). Since T cell inhibition by VACV-219 was observed regardless of co-infection by VTF7-3, we used single infection in our T cell assays. T7-polymerase-independent expression of T7-promoter-driven poxviral genes has been reported before [54]. Recombinant VACV-GFP was provided by Dr. Gary Thomas (University of Pittsburgh School of Medicine, Pittsburgh, PA).

Rabbit polyclonal antisera used in these assays were raised against CPXV219 protein expressed as a glutathione S-transferase (GST) fusion protein in E. coli from a pGEX-3X vector as described [55]. For this construct, primers (Table S1) were used to insert into the pGEM-3X plasmid a BamHI- XmaI linker containing 5' end of the CPXV219 gene in-frame with the GST coding region, and including XhoI and SphI sites into which the remainder of the coding region of CPXV219 was inserted from a XhoI-SphI DNA fragment obtained from p1951.

### Virus titering

BSC40 cells were plated into 6-well plates at 30% confluency. The next day, the cells were infected with 250 µl of a serial 10-fold dilution of the virus preparation or the infected cell lysate. At 1 h p.i., the cells were overlaid with 0.5% agarose (Life Technologies, Grand Island, NY)-EMEM (Quality Biological, Gaithersburg, MD) and incubated for 5 days at 37°C. The cells were fixed with 75% methanol-25% Acetic Acid for 20 min and stained with 0.1% crystal violet -30% ethanol.

### Next generation sequencing of MPXV genomes

Genomic DNA of the wild type MPXV and Δ197 mutant was isolated using DNeasy kit from the virus preparations purified through a 25% to 40% continuous sucrose gradient. DNA libraries were generated by the OHSU Massively Parallel Sequencing Shared Resource (MPSSR) core using the TruSeq DNA Sample Preparation kit (Illumina, San Diego, CA). The sequencing was performed using a MiSeq sequencer (Illumina) at the Molecular and Cellular Biology (MCB) core at the ONPRC. The resulting DNA reads were aligned to the published genome sequence of MPXV-USA2003-039 (GenBank accession # DQ11157). Illumina sequence data were processed using a custom analysis pipeline written by B.N.B. This pipeline has been made available as a module for LabKey Server, an open-source platform for the management of scientific data [56]. The SequenceAnalysis module provides a web-based interface to initiate analyses, manage data, and view results. The source code behind this pipeline is available in a subversion repository (https://hedgehog.fhcrc.org/tor/stedi/trunk/unsupportedModules/labModules/SequenceAnalysis). Raw reads were trimmed by sequence quality using Trimmomatic [57] and aligned against the reference genome using BWA-SW [58]. Single Nucleotide Polymorphisms (SNPs) between reads and the reference sequences were scored with scripts that utilized SAMtools, picard tools (http://picard.sourceforge.net), and bioperl [59], [60].

### Pulse-chase labeling and immunoprecipitation

CHO cells were tranduced with either Ad-tTA (25 MOI) or Ad-197 (20 MOI) and Ad-tTA (5 MOI). At 24 h post transduction (p.t.), the cells were washed with PBS, overlaid with DMEM (Cys^−^/Met^−^), and incubated for 1.5 h. The cells were pulsed with 300 µCi/10^6^ cells for 45 min and the label was chased for the indicated time intervals. CHO cells were washed with ice-cold PBS and lysed with ice-cold PBS-1% NP-40 buffer. Cell lysates were pre-cleared with agarose beads and immunoprecipitated with αFLAG Ab conjugated to agarose beads (Sigma-Aldrich, St. Louis, MO). The samples were eluted from the beads with 50 mM NaOAc-0.15% SDS buffer (10 min, 98°C) and treated with EndoH (Roche Diagnostics, Indianapolis, IN) or PNGase (New England Biolabs, Ipswich, MA) according to the manufacturer's protocols. The samples were separated on a 6% polyacrylamide gel.

### Immunoblot

CHO cell lysates or immunoprecipitated samples were separated on 6% polyacrylamide gels and transferred onto Immobilon PVDF membranes (EMD Millipore, Billerica, MA). The membrane was blocked with 5% skim milk in PBS-0.05% Tween 20 (PBST) buffer and blotted with αFLAG Ab (Sigma-Aldrich, 1∶500) and secondary HRP-conjugated mouse TrueBlot Ab (eBioScience, San Diego, CA) diluted in 5% skim milk-PBST. The immunoblots were developed with SuperSignal West Pico Chemiluminescent Substrate kit (Thermo Fisher Scientific, Rockford, IL).

### Cell-surface biotinylation

CHO cells grown in T75 flasks to 80% confluency were transduced with either Ad-tTA alone (25 MOI) or Ad-197 (20 MOI) and Ad-tTA (5 MOI) or Ad-B22R (20 MOI) and AdtTA (5 MOI). After 24 h incubation, the cells were washed twice with PBS and biotinylated using Pierce Cell Surface Protein Isolation kit (Thermo Fisher Scientific, Rockford, IL) according to the manufacturer's protocol. Biotinylated proteins were immunoprecipitated with NeutrAvidin agarose resin provided with the kit, separated on 6% PAGE gel, and blotted with αFLAG Ab.

### Immunofluorescence and confocal laser scanning microscopy

CHO cells were plated on glass coverslips in 12-well plates at 50% confluency. The next day the cells were transfected with 500 ng of indicated plasmids using lipofectamine 2000 (Life Technologies) according to the manufacturer's protocol. At 24 h p.t., the cells were washed with ice-cold PBS, fixed with 4% paraformaldehyde, and permeabilized with 0.2% Triton X100. The samples were blocked with 2% bovine serum albumin (BSA)-PBS (P-BSA, pH 7.4) and stained with primary mouse αFLAG Ab (1∶1000) and secondary anti-mouse to Alexa Fluor 594 (1∶1000, Life Technologies) diluted in 2% P-BSA. The coverslips were mounted on slides in ProLong Gold antifade reagent with 4,6-diamidino-2-phenylindole (DAPI; Life Technologies) and analyzed with Leica TCS SP laser scanning microscope.

### T cell assays

#### Human and Rhesus Macaque PBMC

T cell responses in PBMC were measured as previously described [21]. Briefly, PBMC were infected with or without the indicated viruses (MOI of 0.3–0.6). After 12 hours of incubation, Brefeldin A (BFA; ICN Biomedicals Inc., Costa Mesa, CA) was added at a final concentration of 2 µg/mL for an additional 6 hours. For αCD3-stimulation, PBMC were infected with or without the indicated viruses (MOI 0.3–0.6) for 12 h prior to incubation with plate-bound αCD3 (0.15 µg/ml, 100 µl/well, clone HIT3a, NA/LE; BD Biosciences PharMingen, San Diego, CA) for 6 h in the presence of BFA. RM PBMC were incubated with soluble αCD3 (0.1 µg/well, clone FN 18) for 6 h in the presence of BFA. The cells were stained overnight at 4°C with Ab specific for CD8β (clone 2ST8.5H7, Beckman Coulter, Brea CA) and CD4 (clone L200, BD Biosciences PharMingen, San Diego, CA). Cells were fixed with 2% formaldehyde in PBS, permeabilized with PermWash (0.1% saponin and 1% FBS in PBS) and stained intracellularly using Ab to IFNγ (clone 4S.B3, eBioscience Inc., San Diego, CA) and TNFα (clone Mab11, eBioscience). Samples were acquired on an LSRFortessa (BD Biosciences) using FACS-DIVA software (BD Biosciences) and analyzed using FlowJo software (Tree Star). Non-viable cells were excluded using a live cell gate based on the viability stain (LIVE/DEAD Fixable Dead Cell Stain, Life Technologies), followed by an optimized lymphocyte gate based on forward and side scatter characteristics. The number of virus-specific IFNγ^+^TNFα^+^ T cells was determined after gating on live CD4^−^CD8β^+^ or CD4^+^CD8β^−^ T cells and subtracting the number of IFNγ^+^TNFα^+^ events from uninfected or unstimulated cultures.

#### Human *Mtb*-specific T cell clones

To study T cell responses in the presence of MPXV 197, BEAS-2b cells were infected with Ad-tTA (8 MOI) or Ad-197 (6 MOI) and Ad-tTA (1.7 MOI) for 72h. Alternatively, to study T cells responses in the presence of CPXV 219 the cells were pretreated with 10 µM ST246 kindly provided by SIGA Technologies (Corvallis, OR) and infected with VACV wild type or VACV-219 recombinant virus (2 MOI, 10 µM ST246) for 2 h. The ST246 drug was included at all other stages of the experiments utilizing VACV infected BEAS-2b cells. Indicated *Mtb*-specific T cell clones were co-incubated with infected BEAS-2b cells for 3h or overnight and then stimulated with peptide CFP10_2-12_ (clone D466 D6), pronase digested Mtb cell wall (clone D160 1–23), or phytohemagglutinin (PHA) in αIFN-γ Ab (clone 1-D1K, MABTECH AB, Nacka Strand, Sweden) coated ELISPOT plates as previously described [25], [26]. The staining was detected with αIFN-γ Ab conjugated to horseradish peroxidase (HRP) clone7-B6-1, MABTECH ABand developed using ABC Vectastain-Elite kit (Vector Laboratories, Burlingame, CA).

#### Rhesus CM9 peptide-specific T cell lines

To study T cell responses in the presence of ST246, HFF cells were pretreated with 10 µM ST246 and infected with indicated viruses (2 MOI, 10 µM ST246) for 2 h. ST246 was included in all subsequent steps of the T cell assay at the same concentration. For the experiments utilizing Ad- MPXV 197, CHO cells were infected with either Ad-tTA (25 MOI) or Ad-197 (20 MOI) and Ad-tTA (5 MOI) for 24 h. The infected cells were overlaid with T cells for specific time periods or overnight. After co-incubation, T cells were collected, washed, and transferred into a fresh plate for stimulation with either autologous BLCL cells pulsed with CM9 peptide (SIVgag181–189, CTPYDINQM; Genscript, Piscataway, NJ) or phorbol 12-myristate 13-acetate (PMA)/Ionomycin in the presence of BFA for 5 h as previously described [23]. The cells were washed with PBS and stained with αCD8 (clone SK1, BD Biosciences) and αCD4 (clone L200, BD Biosciences) Ab and LIVE/DEAD Fixable Dead Cell Stain (Life Technologies) for 30 min at room temperature. The cells were fixed and permeabilized with BD Cytofix/Cytoperm (BD Biosciences) and stained intracellularly with Ab specific to TNFα (clone 6.7, BD Biosciences), IFNγ (clone 25723.11, BD Biosciences), and CD3 (clone SP34-2, BD Biosciences). The samples were analyzed by flow cytometry as described above.

#### Murine T cell assay

Poxvirus-specific T cell responses in murine splenocytes were measured as previously described [20]. Briefly, splenocytes isolated from VACV-infected mice (2×10^6^ PFU/mouse) at 8 days p.i. were stimulated with A20 cells infected with CPXV or VACV (MOI = 5, 16 h) in the presence of Brefeldin A for 6 h. Cells were stained overnight at 4°C with αCD3ε and αCD4 Ab's (clones 145-2C11 and RM 4–5, respectively, BD Biosciences), αCD8 Ab (clone 5H10, Life Technologies), Fc Block (BD Biosciences) and mouse IgG (Sigma). The next day, the cells were washed, fixed, and permeabilized with BD Cytofix/Cytoperm (BD Biosciences) followed by intracellular staining with Ab to IFNγ (clone XMG1.2, BD Biosciences PharMingen) and TNFα (clone MP6-XT22, BioLegend, San Diego, CA). The samples were analyzed by flow cytometry as described above. Non-viable cells were excluded using a live cell gate based on Aqua staining, gated for lymphocytes based on forward and side scatter characteristics followed by gating for CD3ε+. Next, CD3ε+ T cells were gated on either CD4+ or CD8+ and IFNγ+TNFα+ T cells were quantified. Background IFNγ+TNFα+ events from uninfected samples were subtracted. T cell responses to CPXV and CPXV deletion mutants were normalized to VACV.

### Tetramer binding

MaMu-A*01 CM9 tetramer was kindly provided by Marcelo Kuroda (Department of Immunology, Tulane National Primate Research Center). The tetramer was conjugated to Allophycocyanin (APC) using ProZyme PhycoPro GT5 APC kit (Prozyme, Hayward, CA) according to the manufacturer's protocol. Monkey CM9-peptide specific T cells recovered after co-incubation with Ad-197/Ad-tTA or Ad-tTA only infected CHO cells (described above) were incubated with the tetramer for 1 h at 37°C and stained with LIVE/DEAD Fixable Dead Cell Stain (Life Technologies) and Ab specific to CD95 (clone DX2, BD Biosciences), CD28 PE (clone L293, BD Biosciences), CD45 (clone D058–1283, BD Biosciences), CD8 (clone SK1, BD Biosciences), and CD3 (clone SP34-2, BD Biosciences) for 30 minutes at room temperature. The cells were fixed with 2% paraformaldehyde and analyzed by flow cytometry as described above.

### ELISA

Orthopox-specific enzyme-linked immunosorbent assay (ELISA) was performed as previously described [10] using whole-VACV lysate (inactivated by pre-treatment with 3% H_2_O_2_ for 2 h). An internal positive control was included on each plate to normalize between plates and between assays performed on different days. Antibody titers were determined by log-log transformation of the linear portion of the curve, using 0.1 optical density units as the endpoint and performing conversion on final values.

## Supporting Information

Figure S1 **Virus spread into Jurkat T cells is blocked by ST-246.** HFF cells infected with indicated viruses (MOI = 2) were layered with Jurkat T cells at 24 h p.i. After overnight co-incubation, Jurkat T cells were removed, washed, transferred into a fresh plate, and incubated for additional 24 h. The number of infected GFP^+^ cells was measured by flow cytometry.
(TIF)

Figure S2 **A)** **MPXV US2003 recombinant deletion mutant viruses were generated by in-vivo recombination replacing ORFs of interest by an expression cassette for eGFP and GPT.** **B)** Multi-step growth kinetics of MPXV-US2003 and MPXVΔ197. BSC40 cells were infected with indicated viruses at 0.1 MOI. After 30 min of incubation, the inoculum was replaced with growth medium. The cells were incubated for indicated time points, harvested, and used for virus titering.
(TIF)

Figure S3 **NextGen Sequence analysis of MPXV genomes.** Shown is the SNP frequency (>0.5%) compared to a reference sequence. Top: MPXV US2003 compared to US2003-39 sequence in public database (GenBank accession # DQ11157). Bottom: MPXVΔ197 mutant virus compared to predicted sequence.
(TIF)

Table S1 **Primer sequences.**
(DOCX)

Table S2 **Location of SNPs in MPXV analyzed by NextGen sequencing.** Any SNP detected in >1% of reads at that position, with at least 500 reads is shown. For each SNP, the frequency, depth of coverage and predicted amino acid changes are shown. All NT positions are reported relative to the wild-type sequence.MPXV-US2003 did not contain SNPs>1%. All MPXVΔ197 SNPs>1% are located near or within the terminal repeats (NT 1-8836 and 189945-198780) in the intergenic regions.
(DOC)

Table S3 **Skin lesion counts in RM infected with MPXV US2003 wild-type and Δ197 mutant.**
(DOC)
