# Supplementary figures and images for: T Cell Inactivation by Poxviral B22 Family Proteins Increases Viral Virulence
Source: PLoS Pathog. 2014 May 15;10(5):e1004123. doi: 10.1371/journal.ppat.1004123 (PMC4022744; doi:10.1371/journal.ppat.1004123)

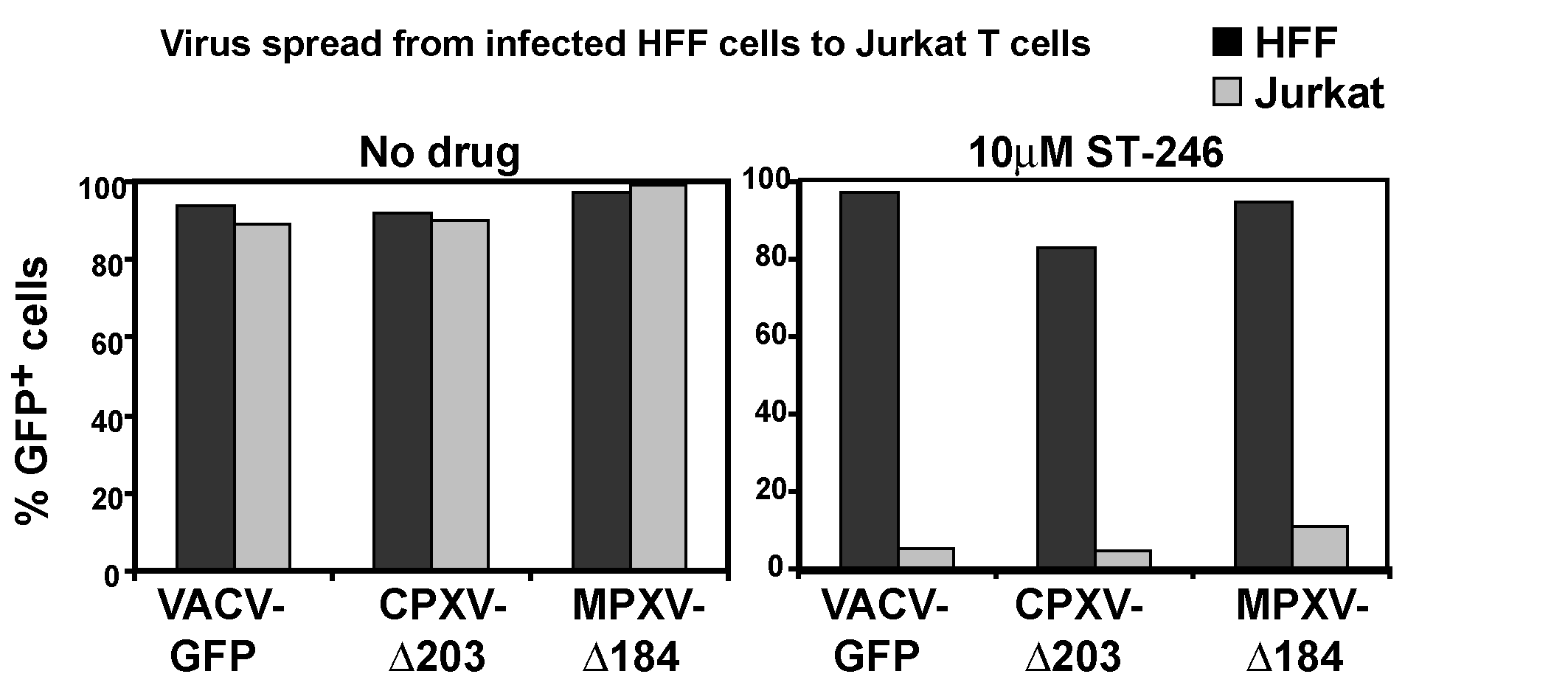

Supplement: Figure S1 — Virus spread into Jurkat T cells is blocked by ST-246. HFF cells infected with indicated viruses (MOI = 2) were layered with Jurkat T cells at 24 h p.i. After overnight co-incubation, Jurkat T cells were removed, washed, transferred into a fresh plate, and incubated for additional 24 h. The number of infected GFP+ cells was measured by flow cytometry. (TIF) [file ppat.1004123.s001.tif]

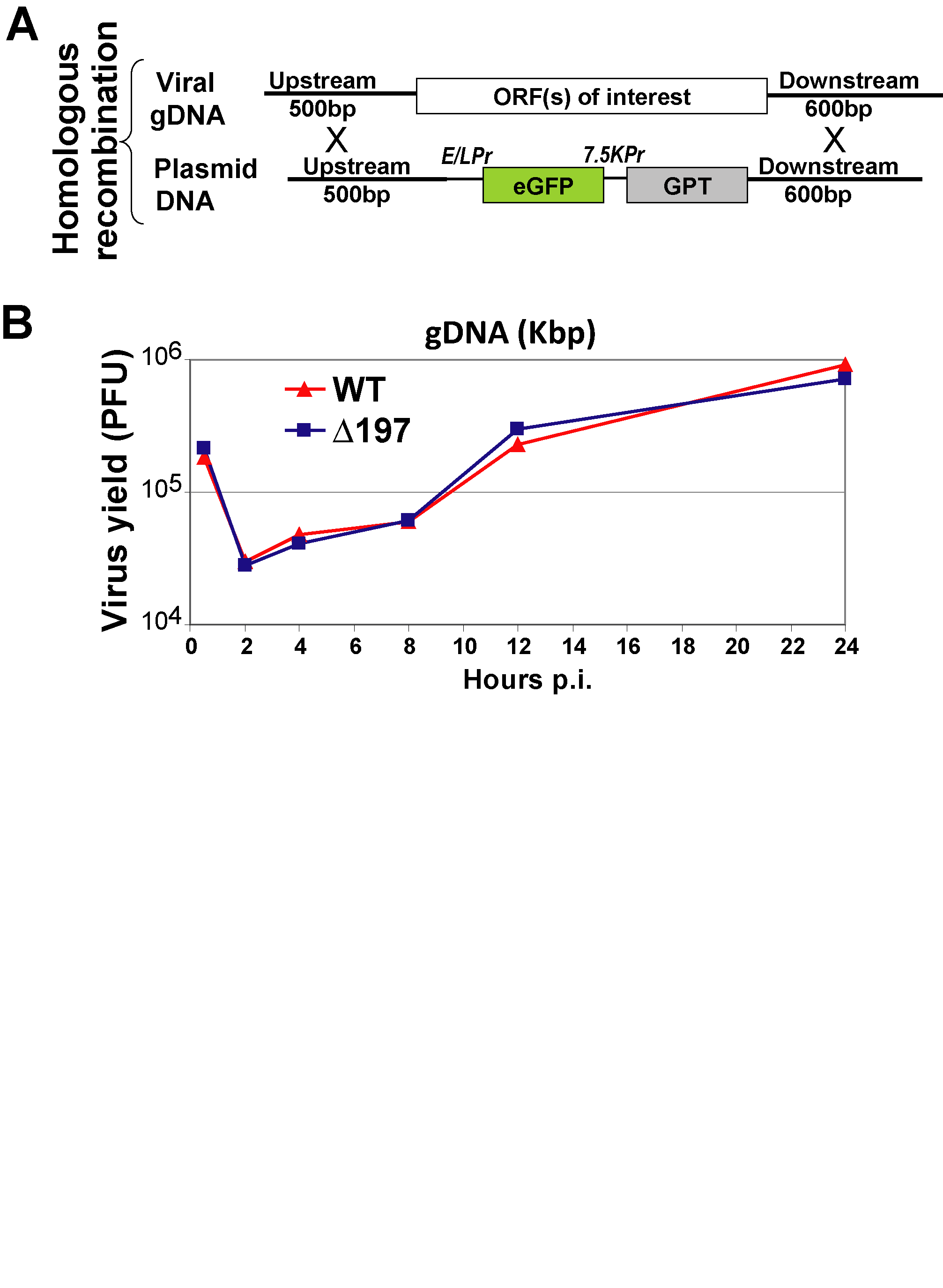

Supplement: Figure S2 — A) MPXV US2003 recombinant deletion mutant viruses were generated by in-vivo recombination replacing ORFs of interest by an expression cassette for eGFP and GPT. B) Multi-step growth kinetics of MPXV-US2003 and MPXVΔ197. BSC40 cells were infected with indicated viruses at 0.1 MOI. After 30 min of incubation, the inoculum was replaced with growth medium. The cells were incubated for indicated time points, harvested, and used for virus titering. (TIF) [file ppat.1004123.s002.tif]

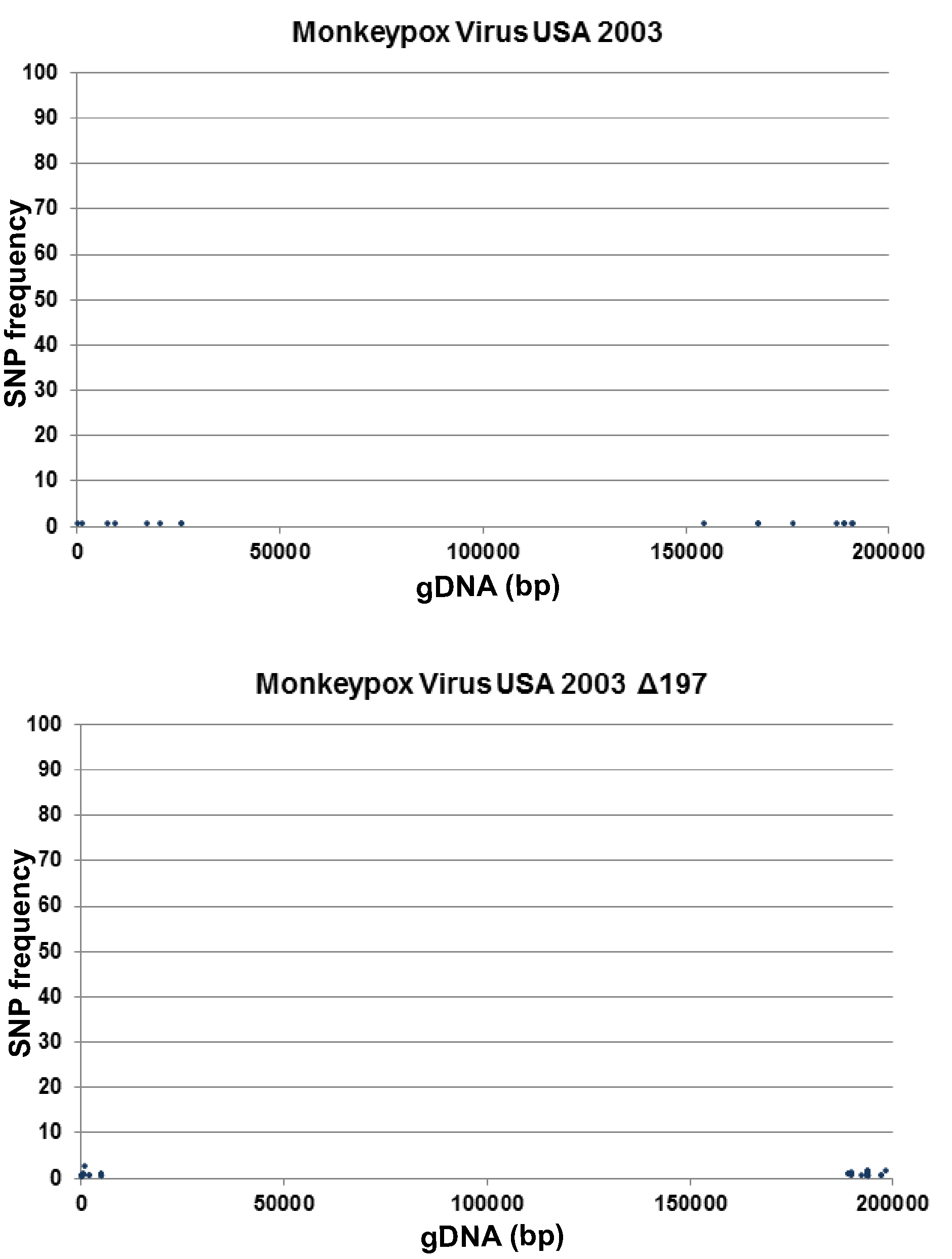

Supplement: Figure S3 — NextGen Sequence analysis of MPXV genomes. Shown is the SNP frequency (>0.5%) compared to a reference sequence. Top: MPXV US2003 compared to US2003-39 sequence in public database (GenBank accession # DQ11157). Bottom: MPXVΔ197 mutant virus compared to predicted sequence. (TIF) [file ppat.1004123.s003.tif]
