# Supplementary material for: T Cell Inactivation by Poxviral B22 Family Proteins Increases Viral Virulence
Source: PLoS Pathog. 2014 May 15;10(5):e1004123. doi: 10.1371/journal.ppat.1004123 (PMC4022744; doi:10.1371/journal.ppat.1004123)
Supplement: Table S1 — Primer sequences. (DOCX) [file ppat.1004123.s004.docx]

**Table S1.** Primer sequences.

| **Primer Name** | **Primer Sequence** |
| --- | --- |
| **MPXV Δ11-25** |  |
| MPXVus8580n-F | GTAGACCTGTAGCCGTCTGTGCTTAATAGAG |
| MPXVus9760-GFP-R | ATCCATATGACTAGTAGATCCTCTAGAGGGAGTCGAATATGGTGTAAATCACAATTGAT |
| MPXVus9760-GFP-F | ATCAATTGTGATTTACACCATATTCGACTCCCTCTAGAGGATCTACTAGTCATATGGAT |
| MPXVus9760-Gpt-R | CTGGAAGAAGATAAACATCACCATAAACAATGTAGAAATCTAGAGCGGATCCGCAGGTTTGC |
| MPVus9760-Gpt-F | GCAAACCTGCGGATCCGCTCTAGATTTCTACATTGTTTATGGTGATGTTTATCTTCTTCCAG |
| MPXVus20700R | AACTAACGCCTACGCCTAAACCCGC |
|  |  |
| **MPXV Δ26-35** |  |
| MPXVus20288n-F | ATCGGATGATGATATCACTGTTTCCAGAGTAG |
| MPXVus21233-GFP-R | ATCCATATGACTAGTAGATCCTCTAGATATTGAATATATAAACCTTTTTACATTCATTATATTATAATTACTTATAGTACTTCAAG |
| MPXVus21233-GFP-F | CTTGAAGTACTATAAGTAATTATAATATAATGAATGTAAAAAGGTTTATATATTCAATATCTAGAGGATCTACTAGTCATATGGAT |
| MPXVus30468-Gpt-R | GCTAACGTAATAATGCGTTATGAAGACACTTATATCATCTAGAGCGGATCCGCAGGTTTGC |
| MPXVus30468-Gpt-F | GCAAACCTGCGGATCCGCTCTAGATGATATAAGTGTCTTCATAACGCATTATTACGTTAGC |
| MPXVus31330-R | AGAGGAGATCAAGGGTTTGGATCAACAGGA |
|  |  |
| **MPXV Δ184-193** |  |
| MPXVus167080-F | ACGTTGTTATGCGTACTACCTGCTGTTGT |
| MPXVus168084-GFP-R | ATCCATATGACTAGTAGATCCTCTAGAGTCACAGGAACAAACTAATACTATAATGGAGATTAG |
| MPXVus168084-GFP-F | CTAATCTCCATTATAGTATTAGTTTGTTCCTGTGACTCTAGAGGATCTACTAGTCATATGGAT |
| MPXVus179413-Gpt-R | ACAACTCAAATTACGATTTCAATATATAATCTTGATGTAATTAGTGTCTAGAGCGGATCCGCAGGTTTGC |
| MPXVus179413-Gpt-F | GCAAACCTGCGGATCCGCTCTAGACACTAATTACATCAAGATTATATATTGAAATCGTAATTTGAGTTGT |
| MPXVus179957-R | TAATCAGTGTTGGGTACGACCGCCT |
|  |  |
| **MPXV Δ194-197** |  |
| MPXVus178592n2-F | GGACGTACACCACTTCATTGCGCT |
| MPXVus179559-GFP-R | ATCCATATGACTAGTAGATCCTCTAGAGATTGCTAATGTTACGTATATCATTTTCGATATCCATGATG |
| MPXVus179559-GFP-F | CATCATGGATATCGAAAATGATATACGTAACATTAGCAATCTCTAGAGGATCTACTAGTCATATGGAT |
| MPXVus188458-Gpt-R  **Primer Name** | ACGAGTAATGAACTGAAATTACAGTACCAAACTGTCTAGAGCGGATCCGCAGGTTTGC  **Primer Sequence** |
| MPXVus188458-Gpt-F | GCAAACCTGCGGATCCGCTCTAGACAGTTTGGTACTGTAATTTCAGTTCATTACTCGT |
| MPXVus 188670-R | TGATAAGCGACGCCATTCATGTCGGA |
|  |  |
| **MPXV Δ197** |  |
| MPVus182428-F | ATCGAGGAGACTGTCTAGAAGCCGTTTATGT |
| MPVusD197-GFP-R | ATCCATATGACTAGTAGATCCTCTAGAGATTGCCGGTCACAAACAAGCCCG |
| MPVusD197-GFP-F | CGGGCTTGTTTGTGACCGGCAATCTCTAGAGGATCTACTAGTCATATGGAT |
| MPXVus188458-Gpt-R | ACGAGTAATGAACTGAAATTACAGTACCAAACTGTCTAGAGCGGATCCGCAGGTTTGC |
| MPXVus188458-Gpt-F | GCAAACCTGCGGATCCGCTCTAGACAGTTTGGTACTGTAATTTCAGTTCATTACTCGT |
| MPVus189027-R | ACTTCGCCGTGGGTGTTAGTTGTCT |
|  |  |
| **MPXV Δ184** |  |
| MPV184-250U-F | CGTGCGCAATTAGATCTAAAGAAGATGTTCC |
| MPV184U-5GFP-R | TGACTAGTAGATCCTCTAGACTTAAAAAATGGTTAGAGCCAAGGGCGTTAAC |
| 5GFP-MPV184U-F | GTTAACGCCCTTGGCTCTAACCATTTTTTAAGTCTAGAGGATCTACTAGTCA |
| 3GPT-MPV184-R | GATTTTTCTAGCCTAATTATTATAAAAAGTATTTTATATCTATCTAGAGCGGATCCGCAGGT |
| MPV184D-3GPT-F | ACCTGCGGATCCGCTCTAGATAGATATAAAATACTTTTTATAATAATTAGGCTAGAAAAATC |
| MPV184-250D-R | CCTATATATCGCATCATCTTGAAAGTCACACAATG |
|  |  |
| **VACV-219 (A625)** |  |
| NcoI-219-5’-SphI-F | CCTGGTACCTCATGAATTTACAGAGATTA |
| NcoI-219-5’-SphI-R | CCGGAATTCCGCATGCCCCAATTGATTGTCATG |
| BssH1- 219-3’- XhoI-F | CCGGAATTCGCGCGCAATAAATACAGAACCAG |
| BssH1- 219-3’- XhoI-R | CCGCTCGAGTACCGATTATCCATAATTTCCATAG |
|  |  |
| **CPXV219-GST** |  |
| CPXV219-GST-F | TCGGGATCCCCATGAATTTACAGAGATTA |
| CPXV219-GST-R | TGCCCCGGGCTCGAGTTCCGCATGCCCCAATTG |
|  |  |
