# Supplementary material for: T Cell Inactivation by Poxviral B22 Family Proteins Increases Viral Virulence
Source: PLoS Pathog. 2014 May 15;10(5):e1004123. doi: 10.1371/journal.ppat.1004123 (PMC4022744; doi:10.1371/journal.ppat.1004123)
Supplement: Table S2 — Location of SNPs in MPXV analyzed by NextGen sequencing. Any SNP detected in >1% of reads at that position, with at least 500 reads is shown. For each SNP, the frequency, depth of coverage and predicted amino acid changes are shown. All NT positions are reported relative to the wild-type sequence.MPXV-US2003 did not contain SNPs>1%. All MPXVΔ197 SNPs>1% are located near or within the terminal repeats (NT 1-8836 and 189945-198780) in the intergenic regions. (DOC) [file ppat.1004123.s005.doc]

**Table S2. Location of SNPs in MPXV analyzed by NextGen sequencing.** Any SNP detected in >1% of reads at that position, with at least 500 reads is shown. For each SNP, the frequency, depth of coverage and predicted amino acid changes are shown. All NT positions are reported relative to the wild-type sequence.MPXV-US2003 did not contain SNPs >1%. All MPXVΔ197 SNPs >1% are located near or within the terminal repeats (NT 1-8836 and 189945-198780) in the intergenic regions.

| **Sample Name** | **NT Position** | **Ref NT** | **Read NT** | **Reads** | **Coverage** | **Percent** |
| --- | --- | --- | --- | --- | --- | --- |
| **MPXV Δ197** | 690 | C | T | 14 | 500 | 2.8 |
| **MPXV Δ197** | 198091 | G | A | 9 | 509 | 1.77 |
| **MPXV Δ197** | 193881 | A | C | 8 | 520 | 1.54 |
| **MPXV Δ197** | 193881 | A | T | 8 | 520 | 1.54 |
| **MPXV Δ197** | 193889 | G | T | 7 | 507 | 1.38 |
| **MPXV Δ197** | 189689 | T | G | 7 | 509 | 1.38 |
| **MPXV Δ197** | 193886 | T | C | 7 | 513 | 1.36 |
| **MPXV Δ197** | 193882 | T | C | 7 | 520 | 1.35 |
| **MPXV Δ197** | 189690 | G | A | 6 | 510 | 1.18 |
| **MPXV Δ197** | 193884 | A | C | 6 | 517 | 1.16 |
| **MPXV Δ197** | 4839 | A | C | 6 | 530 | 1.13 |
