# Supplementary material for: T Cell Inactivation by Poxviral B22 Family Proteins Increases Viral Virulence
Source: PLoS Pathog. 2014 May 15;10(5):e1004123. doi: 10.1371/journal.ppat.1004123 (PMC4022744; doi:10.1371/journal.ppat.1004123)
Supplement: Table S3 — Skin lesion counts in RM infected with MPXV US2003 wild-type and Δ197 mutant. (DOC) [file ppat.1004123.s006.doc]

**Table S3.** Skin lesion counts in RM infected with MPXV US2003 wild-type and Δ197 mutant

| **Days** | **WT-1** | **WT-2** | **WT-3** | **WT-4** | **Δ197-1** | **Δ197-2** | **Δ197-3** | **Δ197-4** |
| --- | --- | --- | --- | --- | --- | --- | --- | --- |
| **7** | 0 | 0 | 20 | 20 | 0 | 0 | 20 | 30 |
| **14** | >100 | >200 | >500 | --- | 10 | 20 | 30 | 30 |
| **21** | 20 | 75 | >> | --- | 5 | 10 | 0 | 0 |
